# Reversible Light-Induced Surface Superwetting of Atomic/Molecular Layer-Deposited TiO_2_:Organic Superlattices for Flexible Optical Applications

**DOI:** 10.1021/acsaom.6c00229

**Published:** 2026-06-18

**Authors:** Lavinia Saltarelli, Ramin Ghiyasi, Anish Philip, Janne-Petteri Niemelä, Ivo Utke, Maarit Karppinen

**Affiliations:** † Department of Chemistry and Materials Science, 174277 Aalto University, Espoo FI-00076, Finland; ‡ Laboratory for Mechanics of Materials and Nanostructures, 34314 EMPA, Swiss Federal Laboratories for Materials Science and Technology, Feuerwerkerstrasse 39, Thun 3602, Switzerland

**Keywords:** atomic/molecular layer deposition, inorganic:organic superlattice thin films, flexible optical materials, superhydrophilic surfaces, mechanical fragmentation

## Abstract

Atomic layer deposition (ALD) is a prominent technique for the precise fabrication of high-quality thin films of various inorganic materials with substantial applications in optoelectronics and semiconductor industries. However, continuous advances are required in materials design to develop next-generation thin-film architectures with multiple functionalities to meet consumer demands. Here, we combine the potential of inorganic ALD with its organic counterpart, molecular layer deposition (MLD), to fabricate TiO_2_-based inorganic:organic superlattice (SL) structures with enhanced mechanical flexibility and tailored optical properties. The sequential deposition of TiO_2_ and monomolecular organic layers through the combined ALD/MLD technique yielded a series of TiO_2_:organic SL thin films with predefined TiO_2_-layer thicknesses ranging between 1 and 10 nm, as confirmed with X-ray reflectivity measurements. We investigate four different organic components, hydroquinone, terephthalic acid, 2-amino-terephthalic acid, and 3,5-pyridinedicarboxylic acid, for their impact on the structural, mechanical, and optical properties of the resultant SL structures. We demonstrate the effects of the different functional groups of the organic precursors on the type of chemical bonding between titanium and the organic moiety and, consequently, on the optical and mechanical properties of the thin films. The TiO_2_:organic SLs outperform the pristine TiO_2_ thin films by presenting excellent resistance to strain-induced fragmentation, increased film flexibility, improved adhesion to the substrate, and exceptional surface wettability, in combination with the possibility of modulating the refractive index—a key feature for their potential application as antireflective coatings. Moreover, we demonstrate reversible light-induced superhydrophilic surface behavior for the TiO_2_:organic SL structures. The TiO_2_-based inorganic:organic SLs we present in this work are expected to open attractive pathways for antireflective coatings with added functionalities such as self-cleaning and antifogging surfaces.

## Graphical abstract

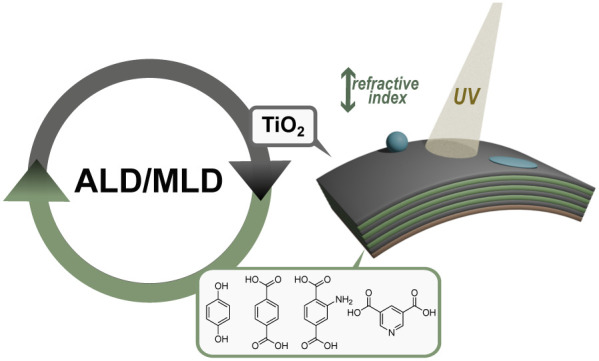

## 1 Introduction

The unmatched material-property portfolio of titanium dioxide has triggered widespread research interest as these properties are highly beneficial for various applications ranging from catalysis and photocatalysis^1,2^ to solar energy conversion,^3,4^ batteries,^5,6^ thermoelectrics,^7,8^ and functional coatings.^9,10^ The high refractive index^11^ and photocatalytic activity,^1^ in addition to the critical-material-free composition, chemical and thermal stability,^12^ and excellent biocompatibility^13^ of TiO_2_ are appealing characteristics that have boosted the research on TiO_2_ both in bulk and thin-film forms,^14^ with several fabrication methods currently implemented in the industrial framework.

Given their optical transparency and high absorption in the UV spectral region, TiO_2_ thin films are especially appealing as antireflective (AR) coatings;^15^ such coatings are already widely applied in optics, solar cells, displays, and contact lenses.^16^ For these applications, the AR behavior is achieved by building multithin-film structures from TiO_2_ and a lower refractive index material, such as SiO_2_, to ultimately attain the destructive interference of light transmitted through the complex structure.^15,17^ Despite the competitive AR performance of these state-of-the-art technologies, further material developments are yet desired for applications requiring, e.g., enhanced mechanical flexibility or specific surface characteristics such as self-cleaning/antifogging functionalities. In particular, the intrinsically rigid structure of conventional crystalline TiO_2_-based thin films, which are plagued with low resistance to strain-induced cracking, is a critical downside for several applications.^18^

A well-established technique for the fabrication of high-quality TiO_2_ thin films is atomic layer deposition (ALD).^14^ In ALD, the thin-film growth is controlled by self-limiting surface reactions between two sequentially pulsed gaseous/evaporated precursors,^19^ which offers unparalleled atomic-level precision and control of film thickness and large-area homogeneity even on complex 3-dimensional or mechanically flexible substrates.^20–23^ These characteristics, in addition to the relatively mild deposition conditions, have made ALD the primary choice in various industrial sectors and an elegant technique for the fabrication of elaborate multilayer structures with specific properties. Moreover, the combination of ALD with its molecular counterpart, molecular layer deposition (MLD),^24,25^ has given rise to various innovative atomic/molecular layer deposition (ALD/MLD) approaches and opened new pathways toward the introduction of organic molecules in an inorganic matrix in thin-film form.^26,27^ This allows us to access the combined properties of the two components—inorganic and organic—with the potential of on-demand designed characteristics related to a distinctive layer architecture. The ALD/MLD technique has already demonstrated its value, for example, for the fabrication of new electrochemically active^28,29^ and light-active^30,31^ metal–organic network materials, as well as layer-engineered inorganic–organic superlattice (SL) structures with tailored functionalities toward, e.g., thermoelectric^32,33^ and magnetic^34^ applications.

Key functional properties of ALD-grown TiO_2_ thin films—such as their optical, mechanical, and surface characteristics—depend on the microstructure and crystal structure of the films.^35,36^ The most common ALD process of TiO_2_, based on TiCl_4_ and H_2_O precursors, yields either amorphous or crystalline anatase- or rutile-structured thin films depending on the thermal range in which the depositions are carried out.^11^ Previous works have moreover demonstrated the successful inclusion of several organic alcohol and amine precursors, such as ethylene glycol, hydroquinone, 4-aminophenol, *p*-phenylenediamine, and 4,4′-oxydianiline, in the TiO_2_ inorganic matrix via ALD/MLD.^37–40^ Most excitingly, for the resulting layer-engineered TiO_2_:organic SLs, properties not readily achieved for conventional TiO_2_-based materials have been demonstrated. For example, the incorporation of hydroquinone and curcumin in ALD-grown TiO_2_ thin films results in the enhancement of visible light absorption toward photocatalytic applications,^41^ triethanolamine promotes the thin film’s visible color change for materials used as chemical separators or in sensing devices,^42^ and 8-hydroxyquinoline enhances the photoluminescence.^43^

In this work, we introduce a new promising strategy for the development of TiO_2_-based functional SL thin film structures with tunable optical and mechanical properties relevant to many advanced applications, including flexible AR coatings with self-cleaning or antifogging surfaces. We investigate an extensive series of TiO_2_:organic thin films fabricated using four commercially available organic compounds compatible with the ALD/MLD methodology, i.e., hydroquinone (HQ), terephthalic acid (TPA), 2-amino-terephthalic acid (A-TPA), and 3,5-pyridinedicarboxylic acid (PDA), and demonstrate that through the proper design of the specific chemical composition and the inorganic–organic layer-piling sequence, we are able to control the film crystallinity, refractive index, and mechanical properties. Most excitingly, we will report for these SL thin films a unique reversible light-induced surface superwetting behavior, relevant for on-demand activation of self-cleaning surface properties.

## 2 Experimental Section

All the ALD/MLD thin-film depositions were carried out at 220 °C in a commercial thermal ALD reactor (Picosun R100). The TiO_2_ layers were deposited through one of the prototype ALD processes^14^ from titanium tetrachloride (TiCl_4_; Merck) as the titanium precursor (pulse/purge: 0.2 s/4 s) and H_2_O as the oxygen source (pulse/purge: 0.2 s/5 s) following the process/parameters established in our previous studies with the same reactor configuration.^44,45^ Both reactants were maintained at room temperature (RT). Nitrogen gas (99.999%; N_2_ 5.0) was employed as a carrier and purge gas in the entire process with regulated flow rates of 200 sccm during the precursor pulsing and purging time. For our reference TiO_2_ thin film, the growth-per-cycle (GPC) was determined to be 0.53 Å/cycle, in line with the GPC values reported in previous studies.^11,14,35,44,45^

For the TiO_2_:organic SL depositions, the ALD (TiCl_4_ + H_2_O) cycles were combined with single MLD (TiCl_4_ + organic) cycles for monomolecular organic layers, following the deposition sequence: *m* × (TiCl_4_ + H_2_O) + *n* × [(TiCl_4_ + organic) + *j* × (TiCl_4_ + H_2_O)]. The organic precursors investigated were: benzene-1,4-diol (hydroquinone, HQ; Reagent Plus, Merck, >99.5%), benzene-1,4-dicarboxylic acid (terephthalic acid, TPA; Merck, 99%), 2-aminobenzene-1,4-dicarboxylic acid (2-aminoterephthalic acid, A-TPA; Merck, 99%), and 3,5-pyridinedicarboxylic acid (PDA; Merck, 98%); molecular structures of these organics together with the corresponding backbone length are shown in Figure 1. For these SL depositions, the organic precursor was loaded in an external heated source at 185 °C for HQ, 220 °C for TPA and A-TPA, and 210 °C for PDA, to ensure optimal sublimation of each organic compound.^46^ For all organic precursors, the pulse length was set to 40 s followed by a N_2_ purge pulse as long as 100 s; these extensively long precursor pulse/purge lengths were selected—based on our experiences on related MLD cycles^27,40,41,47^ —to reach a high level of confidence that the surface reactions got saturated. Here, it should be noted that for the TiCl_4_ + HQ process reported in our previous work, we determined complete saturation curves and revealed that for the HQ precursor, a pulse length of 10 s was enough to reach saturation.^45^

**Figure 1:**
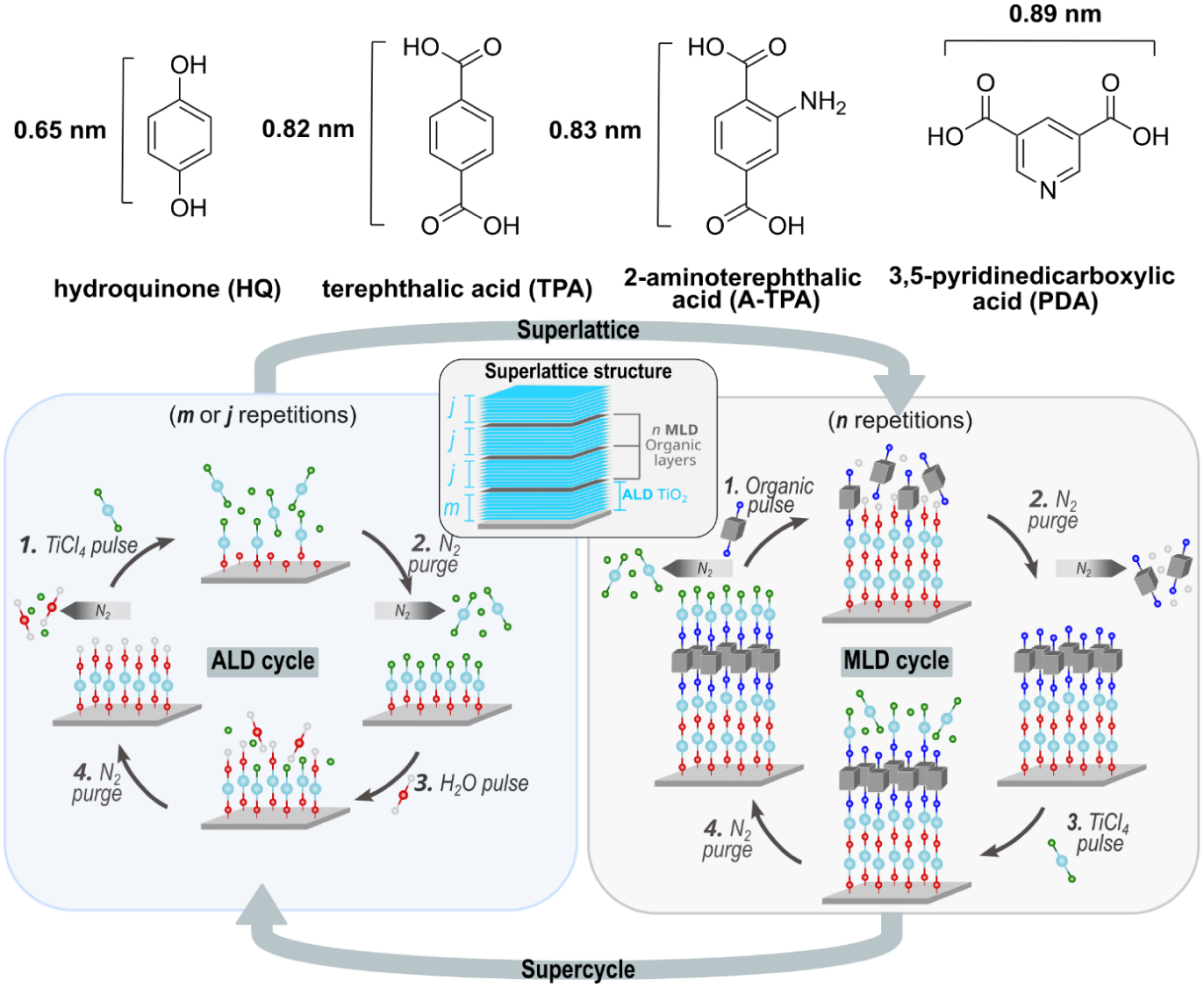
ALD/MLD fabrication scheme for the layer-engineered TiO_2_:organic SL thin films. Top: Names and molecular structures of the organic precursors employed; the backbone lengths given are taken as the oxygen-to-oxygen distance. Below: Generic supercycle methodology for the combination of ALD (TiCl_4_ + H_2_O) and MLD (TiCl_4_ + organics) cycles into TiO_2_:organic SL structures as follows: *m* × (TiCl_4_ + H_2_O) + *n* × [(TiCl_4_ + organic) + *j* × (TiCl_4_ + H_2_O)].

All thin films were simultaneously deposited on silicon (1.0 × 1.0 cm^2^), borosilicate glass (1.5 × 3.0 cm^2^), and polyimide (Kapton 200HN) substrates (4.5 × 4.5 cm^2^ with five precut stripes) to allow for different characterizations. The silicon and borosilicate glass substrates were rinsed in isopropyl alcohol prior to deposition, whereas the polyimide substrates were rinsed in isopropyl alcohol and deionized water and were then placed in the reactor at 280 °C for 1 h to eliminate residual water prior deposition.

A schematic representation of the ALD/MLD process and the resulting TiO_2_:organic SL thin-film architecture is displayed in Figure 1. A fixed total number of cycles, i.e., *m* + *n* [1+ *j*] = 2000 (with *j* + 1 < *m* < *j* + 6 to maintain the total number of cycles equal for all SL sequences), was selected for all thin films, corresponding to a film thickness of approximately 100 nm. To obtain the desired SL thin-film series, the SL repetition (*n*) was varied between 3 and 56 for all four organic precursors. A summary of the deposition sequence parameters, *m*, *n*, and *j*, employed for the fabrication of the four TiO_2_:organic SL sample series with different organics is given in Table S1.

Structural characterization of the thin films through X-ray techniques was performed on a Rigaku SmartLab diffractometer, equipped with a Cu Kα radiation source and a HyPix-3000 detector. Thin film thickness was determined through X-ray reflectivity (XRR) measurements employing a Ge (220) × 2 double-bounce monochromator, which additionally served to confirm the formation of the expected SL structures. Crystallinity and phase composition of the films were investigated using grazing-incidence X-ray diffraction (GIXRD) in a parallel beam mode and an incidence angle of 0.5°. The same measurement conditions and parameters were maintained for all samples.

Vibrational modes deriving from the TiO_2_ and the organic moieties in the films were investigated through Fourier transform infrared (FTIR; Bruker Alpha II) and Raman spectroscopy (Renishaw inVia Qontor with a 532 nm excitation wavelength) for the films deposited on silicon substrates. In FTIR, the interference from the substrate was subtracted from all spectra. UV–vis absorption spectra of the films deposited on borosilicate glass substrates were acquired at wavelengths ranging between 200 and 800 nm in transmission configuration with the front side exposed to the incoming beam with a Shimadzu UV-2600 spectrophotometer, subtracting the contribution of the glass substrate. Optical properties of the TiO_2_ reference film and the SL films with *n* = 56 for each organic component deposited on silicon substrates were determined via spectroscopic ellipsometry (SE) on a HORIBA Uvisel+ spectroscopic ellipsometer using the DeltaPsi2 software. These measurements were carried out in the photon energy range 0.6–6 eV at an angle of incidence of 70°. To model the acquired SE data, Tauc-Lorentz and New Amorphous dispersion formulas were employed for the TiO_2_ layers and the organic layers resulting in optimal fitting with χ^2^ values ≤1. In the cases of SL structures, the New Amorphous dispersion formula was employed for the TiO_2_ layers as well, considering the reduced crystallinity deriving from the inclusion of the organic compounds in the structure, and resulted in optimal fitting. The modeled film architecture consisted of a silicon substrate layer, a 2.5 nm thick SiO_2_ native oxide layer, *n* repetitions of TiO_2_ and organic layers, and a top TiO_2_ layer. The thickness of each layer was estimated from the GPC value and used as an initial guess. Additionally, surface roughness was considered and modeled according to the effective medium theory (EMT). Detailed sample architecture and data from the final fitting results are available in SI, Section III.

Uniaxial tensile testing was performed on a tensile stage (MTI 8000–0010) coupled to a digital optical microscope (Keyence 500F) for *in situ* observation of the fragmentation process in the films deposited on the polyimide substrates (substrate thickness: 50 μm). Mechanical properties were investigated for a TiO_2_ reference film and for the SL films with *n* = 56. A constant strain rate of 1.4 × 10^–4^ s^–1^ by means of displacement control was applied to the tensile stage. For detailed analysis, microscope images were acquired at strain intervals of 1.4 × 10^–4^. Calculated strain values were obtained with digital image correlation following the distance between pairs of points (polyimide features) on the sample surface, with gauge sections set to 5 × 17 mm^2^. The resulting values and respective error margins (standard deviation) are obtained from the measurement of different precut stripes from the same sample, repeated 3–5 times according to the number of available stripes.

Surface wetting properties were investigated with static water contact angle measurements performed at RT on a KSV CAM200 using deionized water (4 μL droplet volume). The contact angle value was determined from the recorded images of the sessile drop, and the values given are averages of three repeated measurements. To regulate the contact angle, the films were first illuminated with UV irradiation under a high-intensity Hg–Xe UV light source (Hamamatsu LC8 spotlight source) with a wavelength of 365 nm and an irradiance of 4500 mW/cm^2^; samples of 1.0 × 1.0 cm^2^ in size were placed in a cylindrical quartz glass vessel with a lamp-to-surface distance of 6 cm. Then, the reverse thermal treatment was carried out in a tubular furnace under a constant nitrogen (99.999%; N_2_ 5.0) flow by heating (1 °C/min) to 100 °C, followed by cooling (1 °C/min) after a 5 min dwell time. In both cases, the contact angle was measured immediately after the treatment, UV or thermal.

## 3 Results and Discussion

### 3.1 Structural Characterization

The ALD/MLD process design allowed us to fabricate with a high level of control the intended TiO_2_:organic SL sample series with all four different organic precursors included in the study: hydroquinone, terephthalic acid, aminoterephthalic acid, and pyridinedicarboxylic acid. These aromatic precursor molecules were selected considering the renowned stiffness of the aromatic core to reduce the possibility of bending and thereby to avoid the unwanted double reactions on the growth surface typically seen for the case of linear organic precursors.^38,40^ Moreover, both the hydroxyl and carboxylic acid reactive groups available in these homobifunctional organics have been previously shown to promptly react with TiCl_4_ to form the Ti–O–R bonding in ALD/MLD.^45^ The small size of the chlorine ligands in the titanium precursor is additionally advantageous as their low steric hindrance favors an efficient growth process, even in the presence of larger organic molecules in ALD/MLD. The resultant Ti-organic bonding is expected to differ according to the reactive groups (hydroxyl or carboxylate) in the organic precursor, resulting in different inorganic/organic interfaces and packing densities of the monomolecular organic layers. Additionally, in two of the organic precursors investigated, there are substituents that could vary the π-electron density/aromaticity of the aromatic rings, i.e., the amine functionality in A-TPA and the pyridine-based ring in PDA.

In Figure 2, FTIR spectra for the TiO_2_ reference film and all TiO_2_:organic SL thin films with the highest number (*n* = 56) of organic layers implemented are displayed. Besides the vibrational modes relative to the Ti–O stretching present in all films, the SL spectra display clear contributions deriving from the introduction of the organic layers in the structure. At the same time, the absence of the characteristic −OH bands confirms that the organic molecules are indeed bonded to the TiO_2_ layers via these groups (i.e., the hydroxyl and carboxylic acid reactive groups in the precursors). As also expected, the spectra show contributions deriving from the aromatic ring for all the SL samples, with the presence of both the aromatic C=C stretching and the out-of-plane C–H bending. Additionally, the spectra of the TiO_2_:A-TPA and TiO_2_:PDA films show, respectively, the presence of the amine stretching mode and the C=N ring stretching vibration, which indicates that these functional groups in A-TPA and PDA are not taking part in the bonding with titanium in the films. For the case of HQ, unidentate bonding of the reactive −OH groups with titanium is the sole possibility,^33,45^ while in the case of the three carboxylic acid-based organic precursors, TPA, A-TPA, and PDA, the carboxylate moieties have in principle multiple possible ways of bonding with titanium. A strong indication of the bonding mode is obtained from the distance Δ (in wavenumbers) between the symmetric (ν_s_) and asymmetric (ν_as_) stretching modes of the carboxylate moiety. For the present TPA, A-TPA, and PDA-based SL films, the Δ values are, respectively, 161 cm^–1^ (1548–1387 cm^–1^), 173 cm^–1^ (1557–1384 cm^–1^), and 166 cm^–1^ (1551–1385 cm^–1^), suggesting a bridging bonding mode for all these cases.^27,48^

**Figure 2:**
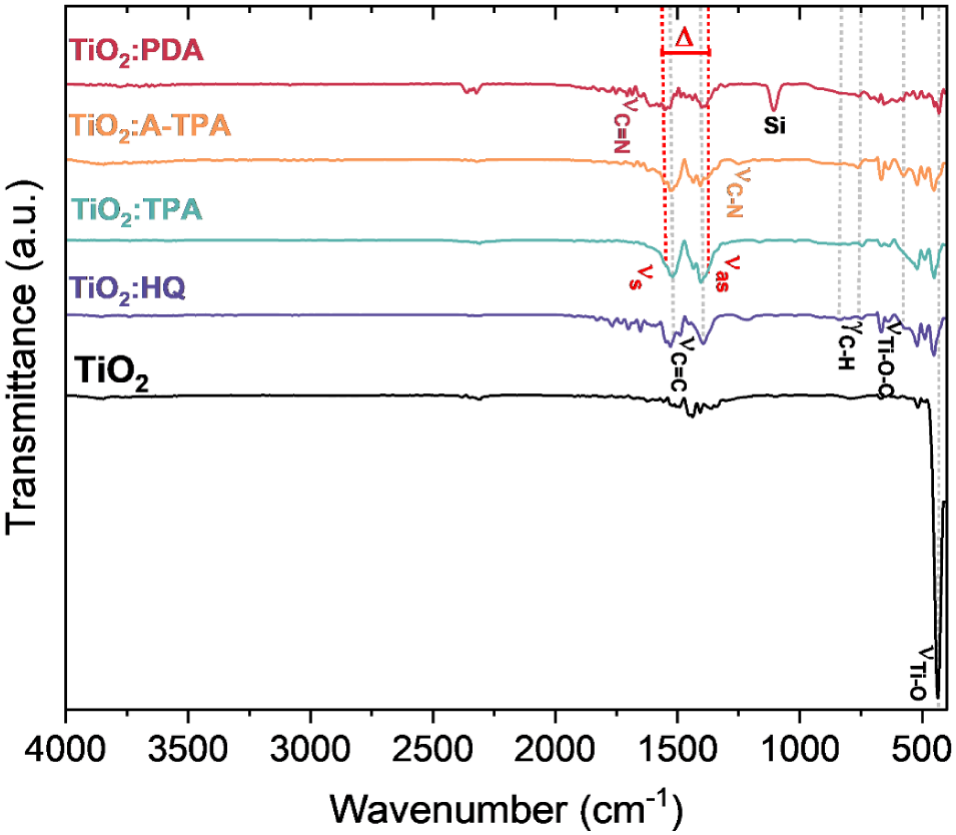
FTIR spectra for TiO_2_:organic thin films with the four different organic precursors (*n* = 56) to reveal the type of Ti-organic bonding; the spectrum of a TiO_2_ (*n* = 0) film is shown for comparison.

When the number of organic layers in the SL structure is decreased from *n* = 56 to 7, the intensity of the FTIR peaks of ν_s_ and ν_as_ stretching modes relative to the carboxylate groups decreases proportionally with *n* (Figure S1). Concurrently, the peak corresponding to the Ti–O vibrational mode increases significantly. Raman spectroscopy was used as an additional tool to probe the chemical state of our SL thin films (Figure S2). For all the four TiO_2_:organic structures, the characteristic Raman vibrations of the aromatic C–C stretching are seen. Additionally, the TiO_2_:A-TPA and TiO_2_:PDA spectra reveal the presence of vibrations relative to the C–O–C moiety. This confirms that the chosen organic precursors are compatible with the ALD/MLD process with no decomposition occurring at the selected deposition temperature and indicates the favorable introduction of the organic layers in the SL structure.

The intended SL structures were verified through XRR, as shown in Figure 3. The regular insertion of organic layers in the inorganic TiO_2_ matrix is seen as the repetition of the Kiessig fringes of higher intensity, i.e., the so-called SL peaks, not seen for the reference (*n* = 0) TiO_2_ film (Figure 3a). From Figure 3b-e, it is clearly seen that the number of lower-intensity fringes between two consecutive SL peaks systematically increases with the increasing number of organic layers: for all the SL films with *n* = 3, 7, 14, and 28, the number of the lower-intensity peaks seen equals to *n*–1, demonstrating that the SL structure is obtained with the correct number of repetitive units as designed from the ALD/MLD process. For the *n* = 56 samples, these weaker peaks also exist but cannot be explicitly counted anymore due to their increasing degree of overlap (Figure 3f).

**Figure 3:**
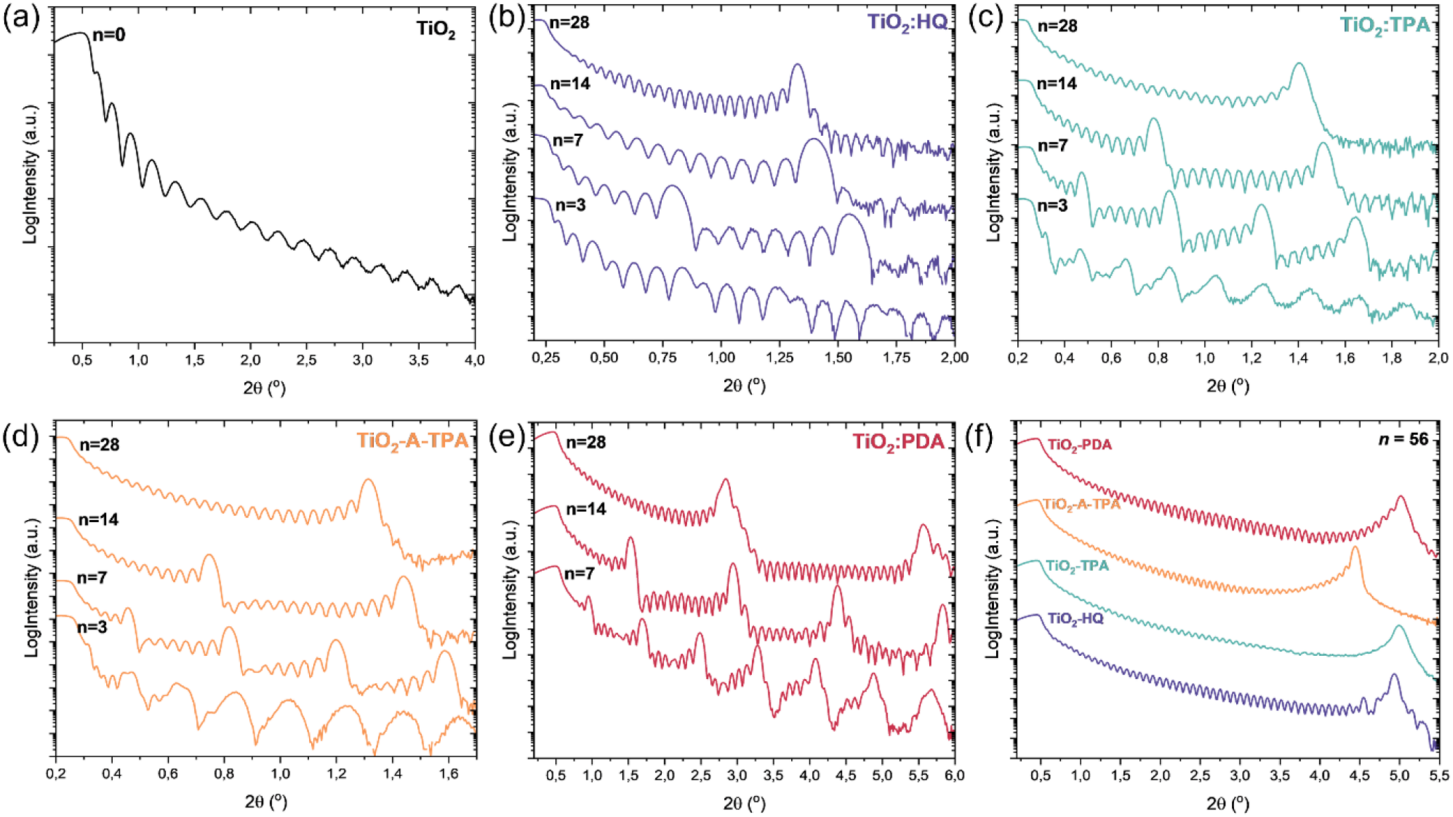
XRR patterns for (a) TiO_2_ (*n* = 0) reference, and (b) TiO_2_:HQ, (c) TiO_2_:TPA, (d) TiO_2_:A-TPA, (e) TiO_2_:PDA thin films with *n* increased from 3 to 28. (f) XRR patterns for TiO_2_:organic (*n* = 56) thin films with the four different organic precursors.

From the XRR data, the overall film thicknesses and also estimates for the individual layer thicknesses could be determined. Detailed information regarding the XRR fitting procedure is found in (SI Section II, Figure S3), and the fitting results are summarized in Table S2. For the TiO_2_ (*n* = 0) reference film, the GPC was found to be 0.53 Å/cycle, as expected. However, for the SL films, we noticed that the TiO_2_ layers were thinner than expected. For example, for the TiO_2_:PDA SL series—as a representative case—the TiO_2_-layer GPC value was found to decrease with increasing *n* as follows: 0.40 Å/cycle for *n* = 7, 0.38 Å/cycle for *n* = 14, 0.32 Å/cycle for *n* = 28, and 0.28 Å/cycle for *n* = 56. Actually, similar observations have been made earlier for several other ALD/MLD-grown inorganic:organic SL systems, although without deeper discussions.^33,40,45,49^ Here, we hypothesize that the reason for this could be that after the MLD pulse the surface is saturated with relatively large organic molecules such that there are significantly less reactive sites for the subsequent ALD growth of TiO_2_ to progress efficiently during the first (TiCl_4_ + H_2_O) cycles. To gain further evidence for this phenomenon, we analyzed the first TiO_2_ layer (grown prior to any organic pulsing) thickness separately and compared it with the rest of the TiO_2_ layer thicknesses for the case of the SL films with *n* = 56 (for which the difference was expected to be the largest). The results for GPC­(1st TiO_2_)/GPC­(TiO_2_ SL) were as follows: 0.45/0.29 Å/cycle for TiO_2_:HQ, 0.63/0.32 Å/cycle for TiO_2_:TPA, 0.37/0.29 Å/cycle for TiO_2_:A-TPA, and 0.62/0.27 Å/cycle for TiO_2_:PDA. The observed trend is clear (and as we expected), despite the small variations in the exact numbers, presumably due to the limited accuracy of the XRR technique.

Fitting of the XRR data also allowed us to determine the thickness and density values of the individual organic layers. First, the average of the individual organic layer thickness for SL thin films (*n* = 7–56) for each of the investigated organic precursors is in reasonable agreement with the expected backbone length (in parentheses): 6.9 Å for HQ (6.5 Å), 9.5 Å for TPA (8.2 Å), 9.7 Å for A-TPA (8.3 Å), and 8.2 Å for PDA (8.9 Å). The organic layer thickness is slightly larger compared to the ideal backbone length of the corresponding molecule for all the other organics investigated except for PDA. Tentatively, we attribute this to the fact that the reactive groups in PDA are not in the *para-*position as is the case for the other three organics; this may possibly lead to a certain degree of tilt in its orientation to achieve bonding through the two carboxylate groups. Moreover, the electron-withdrawing effect of the pyridine ring may also result in a reduction of the carboxylate bond lengths, finally impacting the resulting organic layer thickness value. Most interestingly, the obtained organic-layer densities, i.e., 1.7 g/cm^3^ (HQ), 1.4 g/cm^3^ (TPA), 1.4 g/cm^3^ (A-TPA), and 1.6 g/cm^3^ (PDA), seem to reflect the type of coordination the organic molecules have with the Ti atoms in the surrounding TiO_2_ layers. Namely, while the unidentate coordination of HQ allows the benzene backbones to be more tightly packed, the bridging-type bonding of the carboxylate groups in the TPA-, A-TPA-, and PDA-based films results in the lower density packing. Finally, the slightly denser PDA layer (compared to the TPA and A-TPA layers) is in accordance with the expected tilting of these molecules, as discussed above.

In Figure 4a, GIXRD patterns for selected thin films are displayed; the TiO_2_ film and the SL films with a low content of organic layers in the structure exhibit the characteristic reflections of the tetragonal anatase TiO_2_ crystal structure, similar to the patterns typically reported for ALD TiO_2_ thin films grown from TiCl_4_ and H_2_O in the same temperature range.^14,35,45^ When the number of organic layers is increased (Figure 4b), the degree of crystallinity gradually decreases, and the films with *n* = 28 and 56 appear as essentially amorphous.

**Figure 4:**
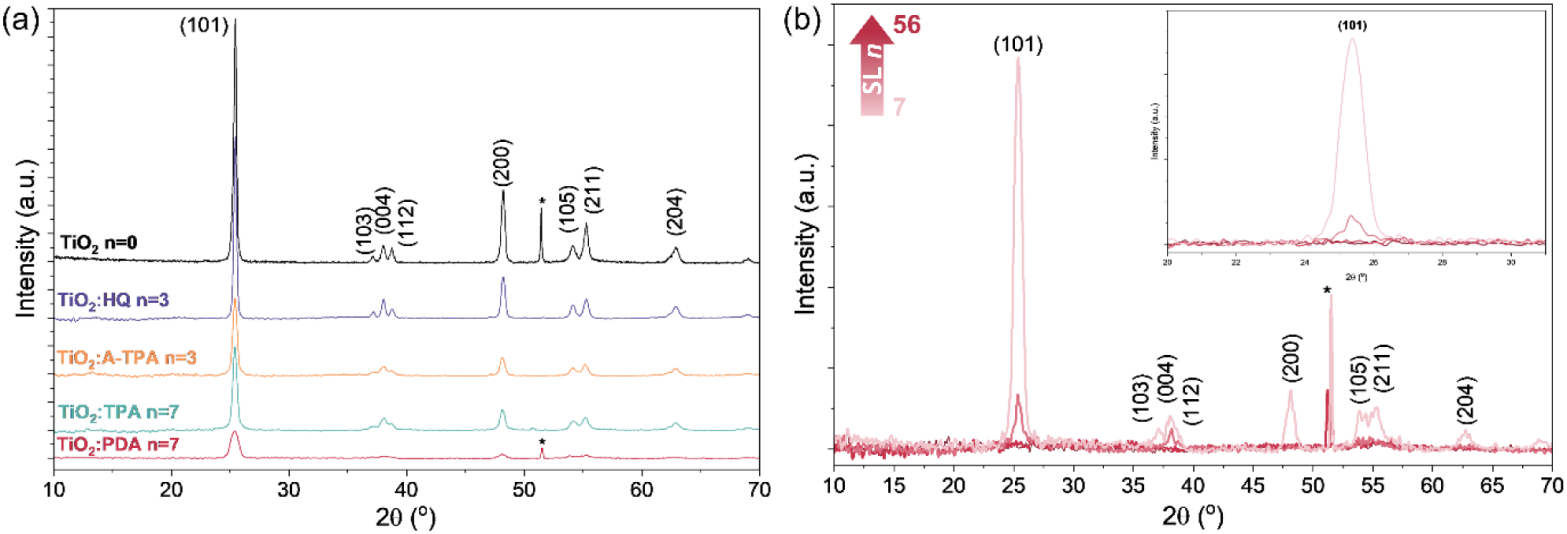
GIXRD patterns of (a) TiO_2_ and representative TiO_2_:HQ, TiO_2_:TPA, TiO_2_:A-TPA, and TiO_2_:PDA thin films (*n* being 3 or 7) displaying the principal reflections of the anatase phase. (b) GIXRD patterns of TiO_2_:PDA thin films with *n* between 7 and 56, showing the gradual loss of crystallinity with increasing *n*; the inset displays the 2θ range of the most intense (101) reflection of TiO_2_ (anatase).

### 3.2 Optical Properties

A combination of UV–vis spectroscopy and spectroscopic ellipsometry was utilized to investigate in detail the optical properties of our TiO_2_:organic films, focusing in particular on the dependence of these properties on the choice of the organic precursor and on the SL architecture. The recorded UV–vis spectra are displayed in Figure 5.

**Figure 5:**
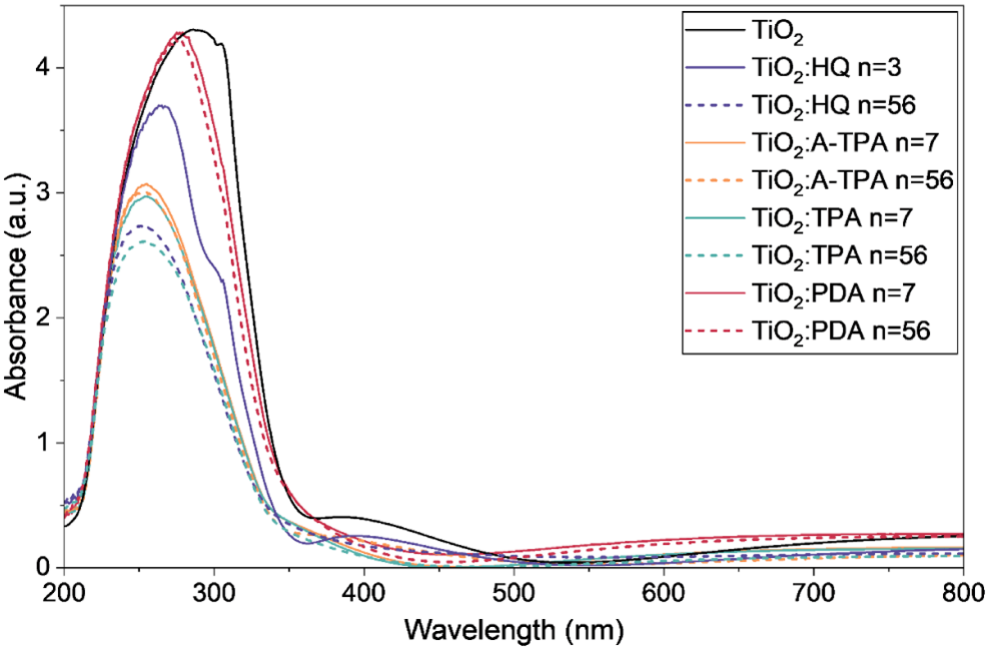
UV–vis spectra for the TiO_2_ reference and for TiO_2_:organic thin films with the four different organic precursors with low (solid line) and high (dashed line) numbers of organic layers in the structure.

The spectrum of the TiO_2_ reference shows the characteristic absorption features of anatase, with an intense absorption peak at 290 nm, ascribed to the band gap electron excitation, and a broad low-intensity absorption band at 390 nm.^50^ The regular introduction of organic layers in the TiO_2_ matrix results in a slight blue shift of the first absorption feature, to 270 nm for TiO_2_:HQ, 257 nm for TiO_2_:TPA, 255 nm for TiO_2_:A-TPA, and 275 nm for TiO_2_:PDA. The increased value for the latter case may derive from the contribution of PDA through the charge transfer band, specifically the electronic transition from the carboxylate group to the aromatic pyridine ring.^51^ For the TiO_2_:HQ films, an additional second absorption feature is observed around 400 nm. In our previous ALD/MLD studies, we observed enhanced absorption in the visible region for amorphous Ti-HQ thin films,^45,47^ and also for TiO_2_:curcumin SL structures;^41^ in the latter case, this was attributed to the strongly visible-light-absorbing organic component, i.e., curcumin. For the TiO_2_:organic SL films here, the increase of *n* results in a minor decrease of the film absorption, possibly due to the loss of crystallinity of the TiO_2_ layers. In this study, we observe—through Tauc plots (Figure S4) and spectroscopic ellipsometry analysis (Figures S5–S6)—a slight band gap widening for our TiO_2_:organic films in comparison to the values of 3.0–3.2 eV reported for crystalline TiO_2_;^52^ the values determined for the pristine TiO_2_ and the *n* = 56 samples are given in Table 1. The optical band gap for the reference TiO_2_ film is well in accordance with reported values. The films with *n* = 56 are predominantly amorphous; thus, the increased band gap value may be affected by both the contribution of the organic component and this significant structural variation.

**Table 1:** Optical Band Gap Values Derived from SE and Tauc Plots for the TiO_2_ and TiO_2_:Organic Films with *n* = 56

	TiO_2_	HQ	TPA	A-TPA	PDA
Optical band gap [eV] (SE)	3.20	3.21	3.35	3.42	3.32
Optical band gap [eV] (Tauc Plot)	3.33	3.38	3.41	3.41	3.34

Spectroscopic ellipsometry allowed us to obtain refractive index and extinction coefficient values for our TiO_2_:organic films (*n* = 56) in the wavelength range between 280 and 1600 nm (Figure 6a and b, respectively). Not surprisingly, we observe a strong dependence of the refractive index on the type of organic component involved. The reference TiO_2_ thin film exhibits a value of 2.513 (λ = 633 nm), consistent with values reported for anatase, whereas all the SL thin films show a substantial reduction of this value below 2, being, respectively, 1.885 for TiO_2_:HQ, 1.585 for TiO_2_:TPA, 1.989 for TiO_2_:A-TPA, and 1.989 for TiO_2_:PDA. For amorphous TiO_2_, the refractive index value is known to be 2.1–2.3;^53^ hence, we believe the significantly low refractive index values observed for our SL films cannot be ascribed solely to the amorphization of the TiO_2_ layers in the SL thin films. In fact, for this study, we had intentionally selected organic components for which we expected lower refractive index values, as our aim was to investigate the role of the organics toward the design of novel TiO_2_:organic compositions suitable for AR materials. The state-of-the-art fully inorganic thin film architectures for AR coatings include the combination of TiO_2_ and SiO_2_ in a multilayer structure. The low refractive index of SiO_2_ (1.46 ^15^) together with the design of the layering sequence, results in the desired reduction of the total refractive index of the multilayered thin film. Optimal AR performance of such compositions has been reported to coincide with refractive indices in the range of 1.2–2.0.^54,55^ Here, in the present study, the refractive index values of the individual organic precursors are quite similar to SiO_2_, being 1.632 for HQ, 1.648 for TPA, 1.547 for A-TPA, and 1.628 for PDA.^56^ As anticipated, the resulting refractive indices for the TiO_2_:organic SL thin films are indeed a combination of the two components, TiO_2_ and organics, demonstrating a substantial contribution derived from the introduction of organic layers into the TiO_2_ matrix. Moreover, as observed in Figure 6c for the case of TiO_2_:PDA and Figure 6d for the case of TiO_2_:TPA, there is a defined SL-structure-dependent variation of the refractive index, which decreases with the increase in the number of organic layers. Further investigation on the effects of varying the SL architecture on the refractive index of the final thin film is anticipated to be beneficial to obtain improved AR performance. However, here we demonstrate the clear and straightforward controllability of this parameter through both the careful selection of the organic precursor and the design of the SL architecture.

**Figure 6:**
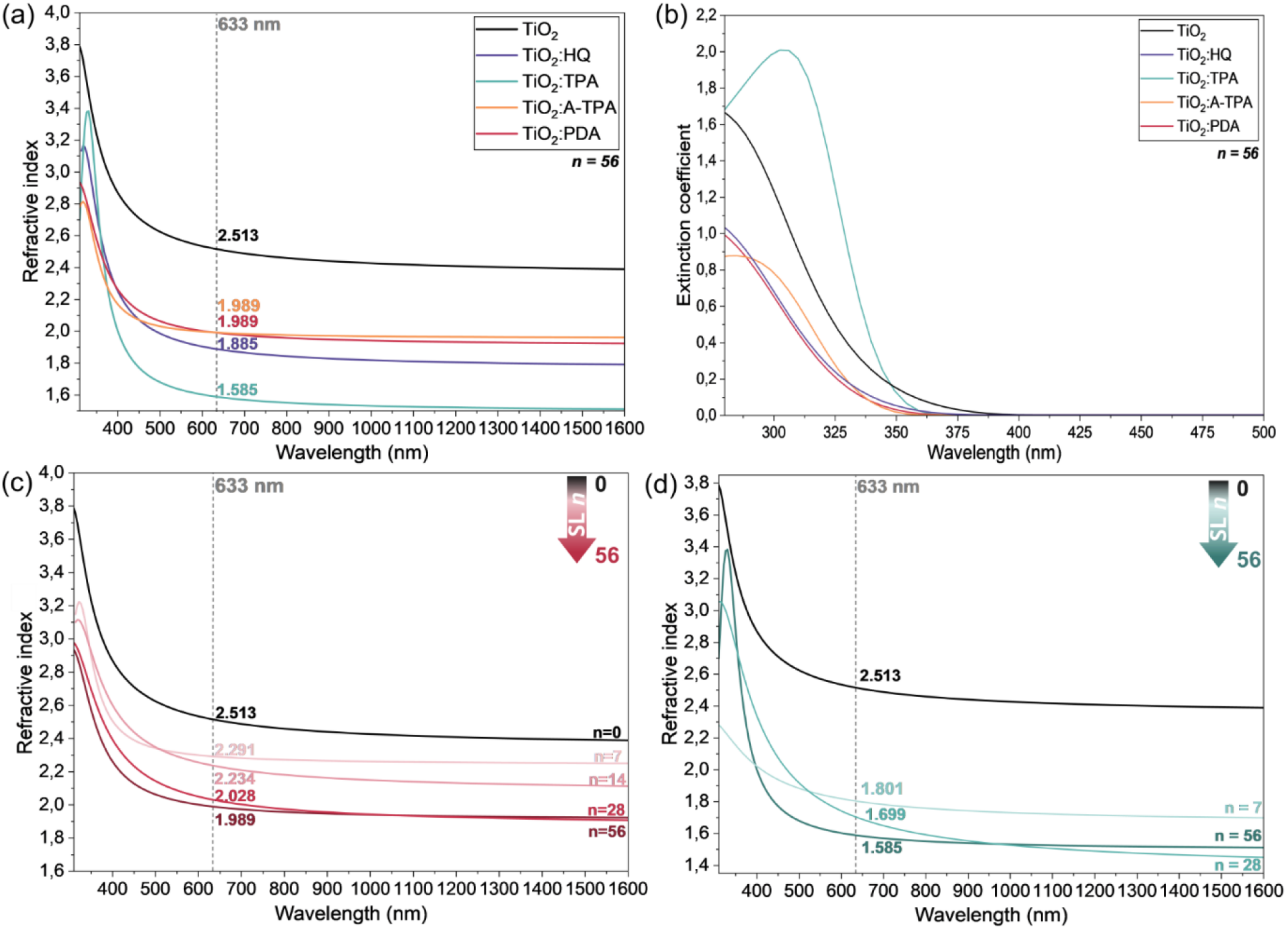
(a) Refractive indices and (b) extinction coefficients of the TiO_2_ reference and TiO_2_:organic thin films (*n* = 56) with the four different organic precursors. Refractive indices of (c) TiO_2_ and TiO_2_:PDA thin films and (d) TiO_2_ and TiO_2_:TPA thin films with increasing *n* from 7 to 56, demonstrating the correlation between the SL architecture and optical properties. In all figures, values of the refractive index at λ = 633 nm are specified next to the relative curve.

### 3.3 Mechanical Fragmentation Properties

The present TiO_2_:organic films were also investigated for their mechanical fragmentation properties considering their relevance for applications requiring flexible and crack-resistant AR coatings. Through uniaxial tensile testing coupled to optical microscopy, we carried out *in situ* studies to determine the crack onset strain (COS) and the critical bending radius (*R*_c_), to obtain information regarding resistance to strain-induced fracture, adhesion to the substrate, and film flexibility.^18,57^ The COS value is defined as the strain value where the first observable crack of finite length perpendicular to the straining axis is seen, while the critical bending radius is obtained through the formula *R*_c_ = (*t*_f_ + *t*_s_)/(2COS), with *t*_f_ and *t*_s_ being, respectively, the thickness of the thin film and of the substrate. Optical microscopy images recorded for the TiO_2_ reference and the TiO_2_:TPA (*n* = 56) thin film subjected to gradually increased values of uniaxial tensile strain are shown in Figure 7 for an exemplary comparison. The COS is clearly higher for the SL thin film compared to the TiO_2_ reference, with the formation of the first crack in the former occurring at an average tensile strain of 0.9% compared to 0.64% for the latter. This substantially increased COS for the TiO_2_:TPA SL film confirms the expected improvement of thin film strain resistance with the introduction of organic layers, in line with our previously reported results for the case of ALD/MLD-grown ε-Fe_2_O_3_:TPA SL structures.^58^ The structure of the chosen organics seems to be critical for the improvement of the mechanical fragmentation properties (Table 2). In fact, the SL with A-TPA, similar in structure to TPA, presents a slightly increased average COS of 0.99%. Contrarily, the pyridine ring and the different position of the reactive groups in PDA appear to reduce the average COS to 0.77% if compared to the other organics. Nonetheless, the combination of TiO_2_ with all the investigated organics is confirmed to substantially suppress mechanical fragmentation with respect to the pristine oxide thin films. Following the same trend, the bending flexibility of the thin film, assessed by the value of *R*_c_, is directly increased with the presence of organic moieties TPA, A-TPA, and PDA in contrast to the TiO_2_ thin film, evidencing the brittle nature of the pure metal oxide,^18^ see Table 2.

**Table 2:** Values of Crack Onset Strain (COS) and Critical Bending Radius (*R*_c_) for the Case of Pure TiO_2_ Thin Films (*n* = 0) and Different TiO_2_:Organic SL Architectures (*n* = 56) on a 50 μm Thick Flexible Polyimide Substrate

	COS (%)	*R*_c_ (mm)
TiO_2_	0.64 ± 0.12	4.0 ± 0.7
TiO_2_:TPA	0.91 ± 0.09	2.8 ± 0.3
TiO_2_:A-TPA	0.99 ± 0.17	2.6 ± 0.5
TiO_2_:PDA	0.77 ± 0.09	3.25 ± 0.4

**Figure 7:**
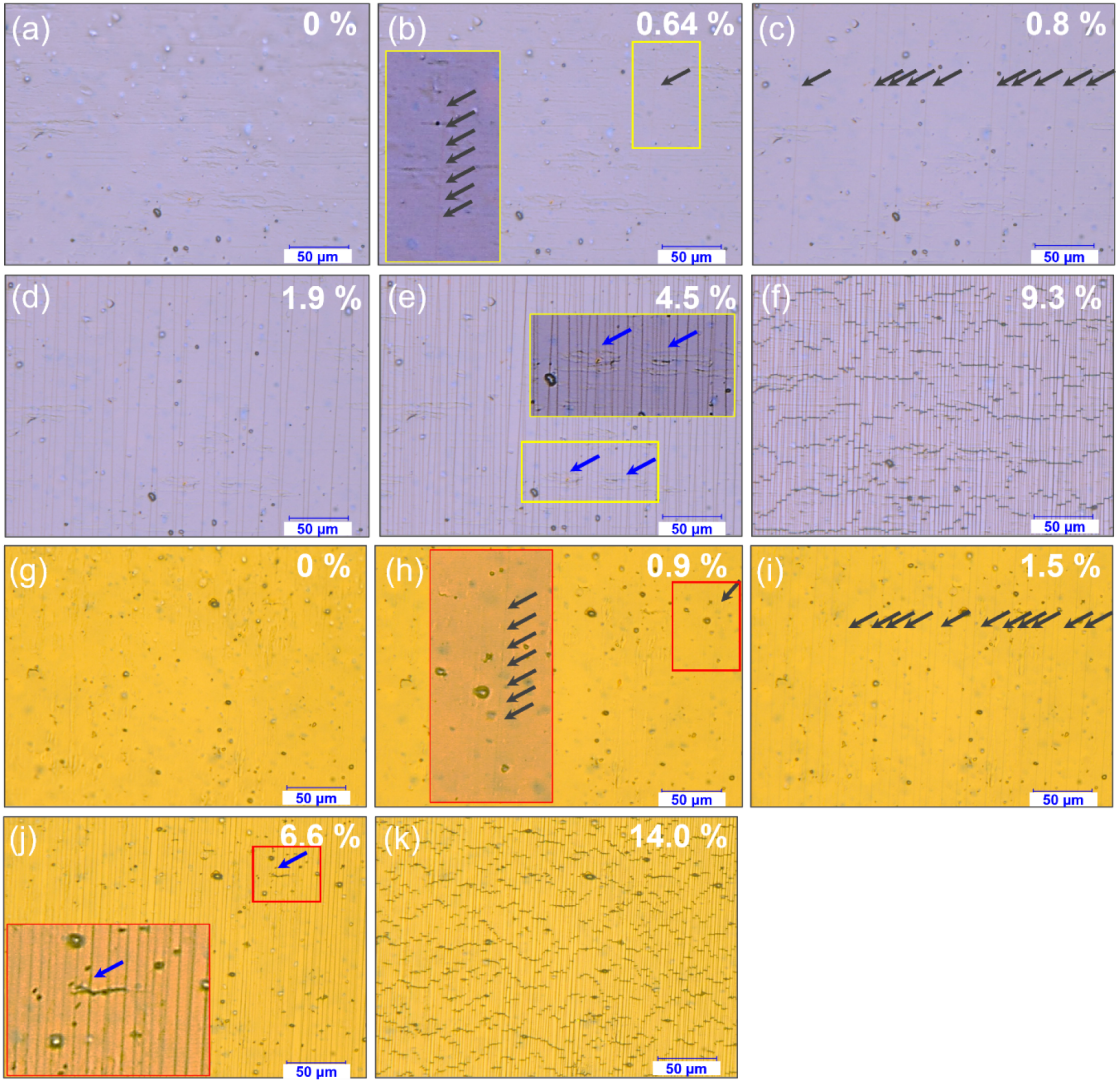
Optical microscopy images recorded *in situ* while uniaxial tensile strain was applied to the films of (a)-(f) TiO_2_ and (g)-(k) TiO_2_:TPA SL (*n* = 56) deposited on polyimide substrates. The applied tensile strain is indicated in the top-right corner of each image. Arrows indicate the crack onset strain (COS; dark gray), the start of transverse buckling and cracking (blue) at nonflat polyimide features. Transverse crack density increases with further increasing tensile strain. Insets show enlarged portions of the image for a better appreciation of the onset features.

When the tensile strain is further increased, the formation of numerous further cracks perpendicular to the direction of the applied strain takes place. The same crack density is observed at a tensile strain value almost a factor of 2 higher for the film with TPA (1.5%), indicating a tougher material than that of the pristine TiO_2_ film (0.8%), see Figure 7. Upon further increase of tensile strain, transverse buckling and cracking are indications of the local delamination of the film, both resulting from the compressive Poisson contraction intrinsically generated in the direction perpendicular to that of the applied tensile strain.^57,59^ The top-view optical observation of transverse contrast features did not allow us to distinguish between these two transverse stress-releasing mechanisms. It was found that such contrast was first formed at nonflat polyimide substrate features already visible at zero strain. Tensile strains for transverse contrast features denoting delamination appeared at strains of 6.6%, 6.0%, and 4.5% for TiO_2_:TPA, TiO_2_:PDA, and TiO_2_ films, respectively. The inorganic:organic SLs thus showed improved film adhesion resisting to higher strains than the inorganic pristine oxide film. For several functional coatings, especially in AR applications that require a certain degree of curvature, the brittleness of the oxide-based thin films and the consequent cracking upon bending drastically degrade the physical properties.^60,61^ Widespread efforts are being directed toward architectural design to improve the flexibility of such films, which nonetheless display intrinsic limitations.^62^ Here, we demonstrate that the inclusion of sequential organic layers in an inorganic metal oxide matrix is a promising solution to reduce this disadvantageous rigidity toward higher-performance functional coatings of this kind. From our analysis, we evidence the optimal mechanical properties of benzene ring-based organic precursors with para-positioned reactive groups for their inclusion in the TiO_2_ matrix. While the use of PDA still leads to an improvement of the COS compared to the pristine oxide, the tilted orientation of the pyridine-ring-based compound in the organic layer, possibly caused by the reactive groups placed in meta positions, results in a lower resistance to crack-induced strain than the other organics. Nonetheless, the high density of the organic layers, a direct consequence of the described structural features, leads to SL films with high adhesion to the underlying substrate. We thus can corroborate that, in line with the results from the previous sections, the choice of the organic molecular structure is crucial toward its defined orientation in the SL. Consequently, the structural arrangement and the organic layer density are determining factors that directly control the mechanical fragmentation properties, the film flexibility, and the quality of the film-to-substrate adhesion.

### 3.4 Modulation of Surface Wettability

Previous studies have reported the possibility to modify the surface wettability of TiO_2_ thin films with UV irradiation.^15,63,64^ Here, we systematically investigated the effects of the monomolecular organic layers embedded within the TiO_2_ films on this wettability behavior. This also allowed us to address the possible detrimental consequences of the UV irradiation on the inorganic–organic thin films in general. For these measurements, the UV irradiation time was initially set to 10 min, and the films (*n* = 56 samples) were studied through water contact angle measurements. All the as-deposited samples were hydrophilic, but the contact angles were higher for the SL films (varied between 65° and 80°) compared to the TiO_2_ reference (45°), see Figure 8. Surface roughness is known to increase the contact angle;^65^ however, this is not the reason here as according to the XRR data fitting, the amorphous *n* = 56 SL thin films were appreciably smooth (roughness below 0.2 nm), in line with previous results for other amorphous inorganic:organic SL structures.^66^ We therefore propose that the underlying organic layers play a crucial role in the thin film surface behavior; especially important could be the polarity of these organic layers controlled by the electronic effects of the functional groups. Molecular dynamics studies have demonstrated the substantial effects of surface polarity on the wettability of rutile TiO_2_.^67^ We tentatively suggest that in our TiO_2_:organic thin films, electron density is shifted toward the underlying organic layer which leads to a lower surface polarity, meaning an increased hydrophobic behavior compared to the case of the TiO_2_ reference.

**Figure 8:**
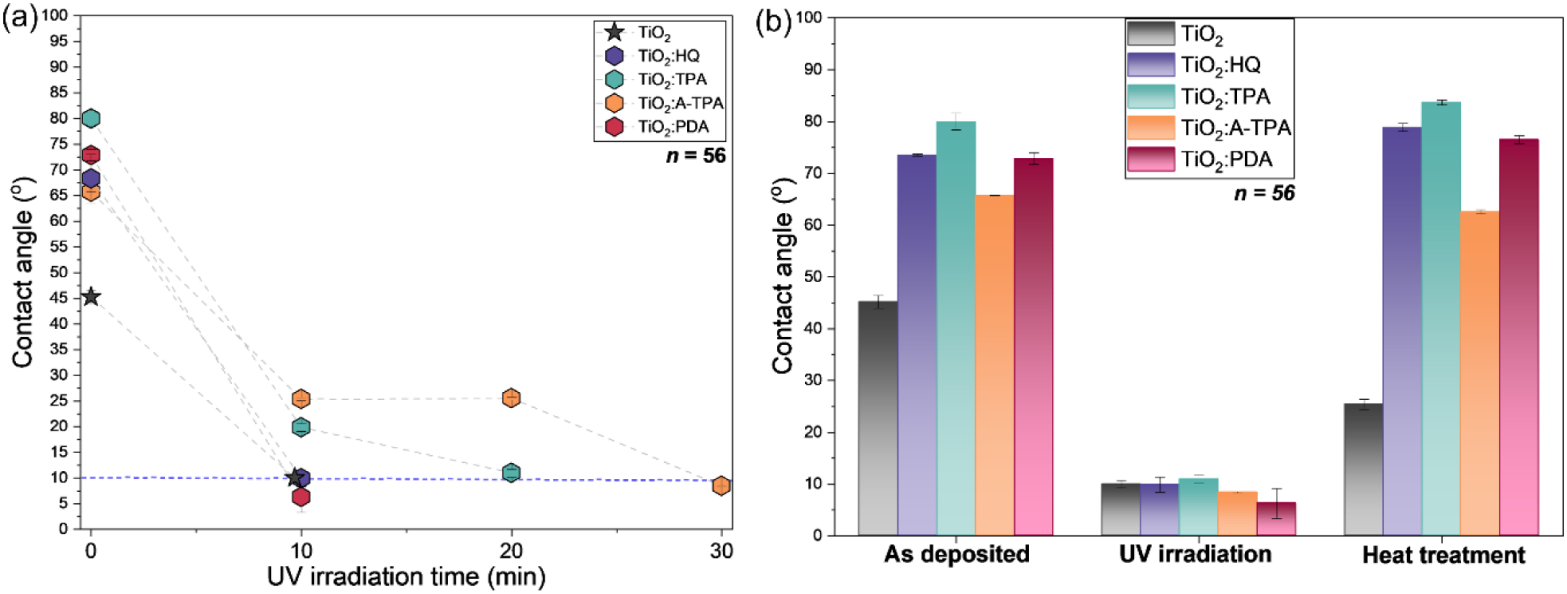
(a) Water contact angle data for TiO_2_ (solid star symbol) and TiO_2_:organic (solid hexagonal symbols) thin films following UV irradiation of 0 min (as deposited) and 10 min, with an increase of this value to 20 and 30 min for the TPA and A-TPA films, respectively. The dashed blue line indicates the contact angle value of 10°, below which the thin film surface is defined as superhydrophilic. (b) Water contact angle data for TiO_2_ and TiO_2_:organic thin films as deposited, following UV irradiation (corresponding to the final irradiation time for each film), and after thermal annealing at 100 °C, demonstrating the clear reversible character of the superhydrophilic surface behavior.

Upon the 10 min irradiation under UV light, the contact angle decreases remarkably for all the thin films studied (TiO_2_ and TiO_2_:organic); for the TiO_2_:PDA film, the contact angle goes clearly below the superhydrophilicity limit of <10°, and for the TiO_2_:HQ and TiO_2_ films too, the 10° limit is reached. For the TiO_2_:TPA and TiO_2_:A-TPA films, a longer UV radiation time, 20 and 30 min, respectively, was needed to make them superhydrophilic (Figure 8a).

Clarifying the mechanism behind the switchable surface hydrophilic behavior is out of the scope of this study. However, in previous literature for TiO_2_, this behavior has been attributed to the generation of surface hydroxyl groups upon the UV exposure conducted in air.^63^ We therefore investigated the possibility of removing any newly generated −OH groups through a mild thermal treatment on the TiO_2_:organic SL thin films. Figure 8b displays the exciting results of the contact angle measurements, clearly showing the reversible effect on the UV-irradiated superhydrophilic surface following a heat treatment at 100 °C, with the contact angle after annealing returning to the original values of the as-deposited films. Additionally, it must be noted that for the TiO_2_ thin film, the annealing process is not sufficient to regenerate the as-deposited surface, with the contact angle after heat treatment reduced to approximately half (25.3°) of the original one (45.2°). Recorded images for TiO_2_ and all TiO_2_:organic SL thin films after each step are displayed in Figure S7. Note that considering the high intensity of the UV irradiation applied, XRR patterns were acquired for each of the investigated SL thin films to confirm that the structure and SL architecture remained unaffected (Figure S8). The thin films of TiO_2_:HQ, TiO_2_:TPA and TiO_2_:PDA exhibit identical patterns to the as-deposited ones, demonstrating no adverse effects of the irradiation process on the monomolecular organic layers. However, we must note the slight modification of the XRR pattern for the case of A-TPA equivalent to a minor thickness decrease of 0.5 nm, possibly due to the excessive UV exposure. XRR patterns have been additionally acquired following the heat treatment (Figure S9) and demonstrate the excellent thermal stability of the ALD/MLD-grown SL architectures as no variation compared to the pattern following the UV irradiation is observed.

The UV-light-switchable and reversible superhydrophilic surface of our ALD/MLD-fabricated TiO_2_:organic SL thin films is expected to be especially appealing for applications related to AR coatings in photovoltaic (PV) panels. Such coatings require self-cleaning surfaces capable of removing dust particles or water droplets common in outdoor applications, in order to maintain the high PV performance when the device is exposed to the environment.^68^ Conventionally, the self-cleaning coating is applied as an additional layer, generally employing transparent polymer-based films that are fabricated to be highly hydrophobic or hydrophilic.^69,70^ Here, we believe the present TiO_2_:organic coatings could open the possibility of having two self-cleaning behaviors in the same protective coating, with a facile switching and reversible character between superhydrophilic and low-wettability surfaces. These SL thin films additionally display AR properties, thus reducing the number of required layers to obtain the desired characteristics and, consequently, the total device fabrication cost.

## 4 Conclusions

We have fabricated new types of TiO_2_-based inorganic–organic superlattice thin films with four different organic precursors via ALD/MLD. A simple deposition process design allowed us to obtain varied superlattice architectures in which monomolecular organic layers are sequentially embedded in nanostructured inorganic TiO_2_ stackings. This method results in stable and homogeneous thin films with structural and optical properties directly related to the designed composition and layer sequence, demonstrating its great potential for the fabrication of novel functional coatings.

Detailed structural characterization verified the desired SL structure in all tested organic precursors. The chemical bonding between the Ti atoms and organic molecules is dictated by the reactive and other functional groups of the specific molecule and leads to consequential variations in the SL structure, such as organic layer thickness and density, according to the organic packing. Harnessing this knowledge, we were able to control the optical properties, specifically the refractive index, through both the choice of the organic precursor and the engineering of the nanostructure, thus evidencing the strong structure–property relationship in the SL thin films grown via ALD/MLD.

Moreover, we revealed superior mechanical fragmentation properties for the ALD/MLD-grown TiO_2_:organic SLs, which overcome the rigid structure of pristine TiO_2_ films presenting enhanced film flexibility, reduced crack formation upon the application of tensile strain, and improved adhesion to the substrate. Additionally, we investigated the surface hydrophilic behavior and demonstrated the UV-light-switchable superhydrophilicity of the TiO_2_:organic thin-film surfaces. The surface wettability properties are fully reversible for all TiO_2_:organic SLs following a mild thermal treatment, contrary to the TiO_2_ thin films. This unique feature adds to the excellent thermal stability displayed by SL structures. A comprehensive summary of the functional properties exhibited by the ALD/MLD-fabricated SL thin films is found in Table S3.

Overall, we believe that the mechanically flexible TiO_2_:organic SL thin films presented in this work are strong candidates for many next-generation applications of advanced functional materials, specifically opening new pathways for AR coating design through the tunability of the refractive index directly correlated to the SL architecture. The ALD/MLD approach is thus pivotal for the development of new AR material compositions which, as discussed, overcome the disadvantageously rigid fully inorganic structures. Moreover, the excellent superhydrophilic surface performance suggests additional possibilities of SL thin films for self-cleaning or antifogging surfaces. We are thus convinced that this work may inspire novel thin-film architectures based on the concept of on-demand combination of functional properties of each individual component for the fabrication of novel high-performance thin-film materials.

## Supplementary Material



## Notes

Additional details on structural and optical characterization of the TiO_2_:organic thin films SL structures, including Raman spectra, XRR data fitting and analysis, SE data fitting, and surface wettability studies (PDF)
