# Supplementary material for: Reversible Light-Induced Surface Superwetting of Atomic/Molecular Layer-Deposited TiO2:Organic Superlattices for Flexible Optical Applications
Source: ACS Appl Opt Mater. 2026 Jun 18;4(7):2083–95. doi: 10.1021/acsaom.6c00229 (PMC13411043; doi:10.1021/acsaom.6c00229)
Supplement: Supplementary file 1 [file ot6c00229_si_001.pdf]

Supporting Information

Reversible light-induced surface superwetting of atomic/molecular layer deposited TiO_2:organic superlattices for flexible optical applications

*Lavinia Saltarelli,[1] Ramin Ghiyasi,[1] Anish Philip,[1] Janne-Petteri Niemelä,[2] Ivo Utke,[2] Maarit Karppinen[1]**

[1]*Department of Chemistry and Materials Science, Aalto University, Espoo FI-00076, Finland*
[2]*Laboratory for Mechanics of Materials and Nanostructures, EMPA, Swiss Federal Laboratories for Materials Science and Technology, Feuerwerkerstrasse 39, Thun 3602, Switzerland*

**Corresponding Author: Maarit Karppinen,* maarit.karppinen@aalto.fi

Table of Contents

Section I. *ALD/MLD deposition sequence design*...page S3

Section II. *Thin film structural characterization*...page S4

Section III. *Optical properties of TiO_2:organic SL thin films*..............................page S7

Section IV. *Analysis of surface wettability of TiO_2:organic SL thin films*..............page S10

Section V. *Summary of functional properties of TiO_2:organic SL thin films*..............page S13

Section I. *ALD/MLD deposition sequence design*

$TiCl_4$ pulse (s)	$TiCl_4$ purge (s)	H_2O pulse (s)	H_2O purge (s)	Organic pulse (s)	Organic purge (s)	*m*	*n*	*j*
0.2	4	0.2	5	40	100	2000	0	0
						500	3	499
						250	7	249
						138	14	132
						68	28	68
						40	56	34

Table S1. Deposition sequence and pulse/purge length parameters employed for the TiO_2 reference and the TiO_2:organic SL thin films.

Section II. *Thin film structural characterization*

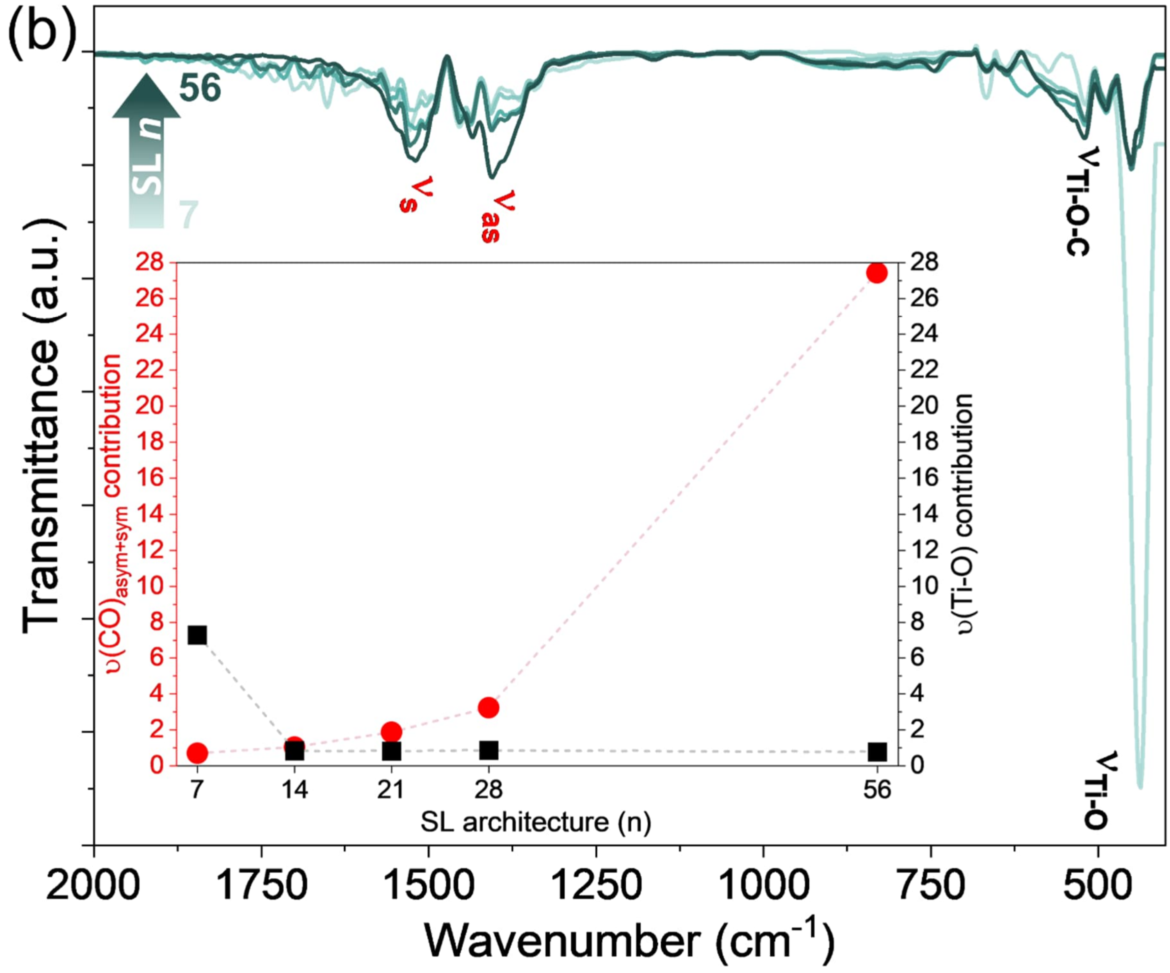

Figure S1. FTIR spectra of TiO_2:TPA SL thin films with *n* varying between 7 and 56. The intensities of the peaks deriving from the ν_s and ν_{as} stretching vibrational modes of the carboxylate group increase with increasing *n*, while the peak of the Ti-O vibrational mode is most intense for low values of *n*. The fitted peak areas of the specified peaks (shown in inset) confirm the described tendency.

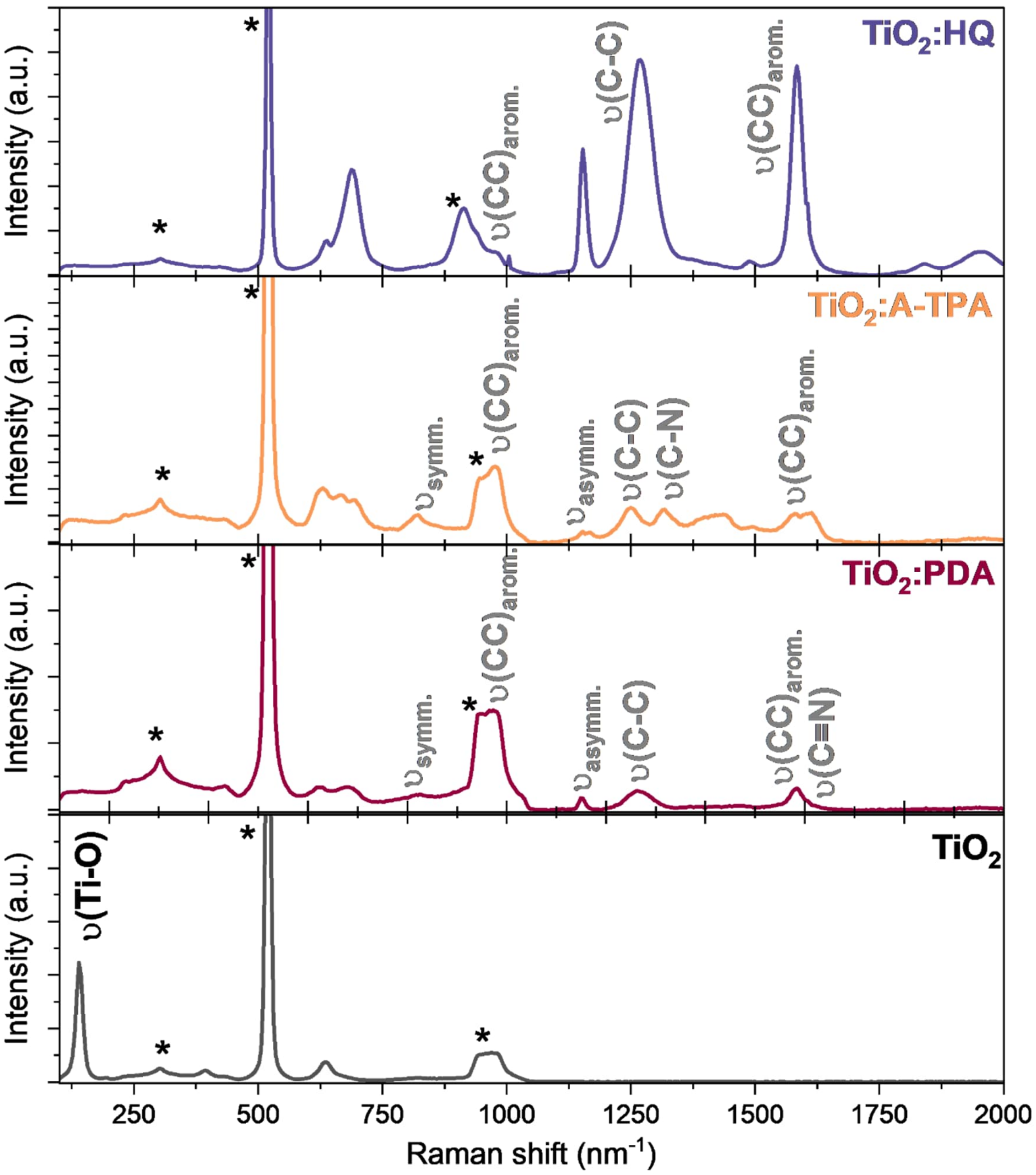

Figure S2. Raman spectra of TiO_2 reference and TiO_2:organic SL thin films with n = 56. All SL thin films display characteristic vibrational modes deriving from the aromatic ring; the TiO_2:A-TPA and TiO_2:PDA films show additionally the presence of the symmetric (ν_s) and asymmetric (ν_{as}) vibrational modes due to the carboxylate moiety. Moreover, the TiO_2:A-TPA film exhibits contribution deriving from the C-N stretching of the amine moiety, whereas a peak deriving from the C=N ring stretching vibration is present in the spectrum of the TiO_2:PDA film. Compared to the spectrum of the TiO_2 reference, the SL spectra lack a vibrational mode due to Ti-O, possibly due to the amorphous nature of the TiO_2 layers in these films. Substrate (silicon) contributions are indicated by an asterisk (*).

XRR data fitting procedure

To obtain the layer characteristics of the TiO_2:organic SL thin films, a fitting procedure for all XRR patterns was carried out. The initial parameters are obtained from theoretical values (i.e., organic backbone length, thickness calculated from the GPC if the TiO_2 reference and calculated according to each SL deposition sequence). A reduced 2θ range is selected coinciding with the range in which the SL fringes are clear, as displayed in Figure S2. The model for the SL architecture is built in the XRR fitting program by indicating the substrate and repetition of individual TiO_2 and organic layers considering the deposition sequence and indicating the value of the following parameters for each layer: theoretical thickness, density, and roughness. Initially, the simulation margins for each parameter were set to ±30% of the theoretical value, then reduced after achieving sequential repetitions of error-free fitting. Each parameter is finally fit individually to optimize the final value. The process is terminated when all the parameters are optimized (below 5% simulation margin), the shape of the fitting curve matches with the acquired XRR pattern, and the χ^2 is minimized.

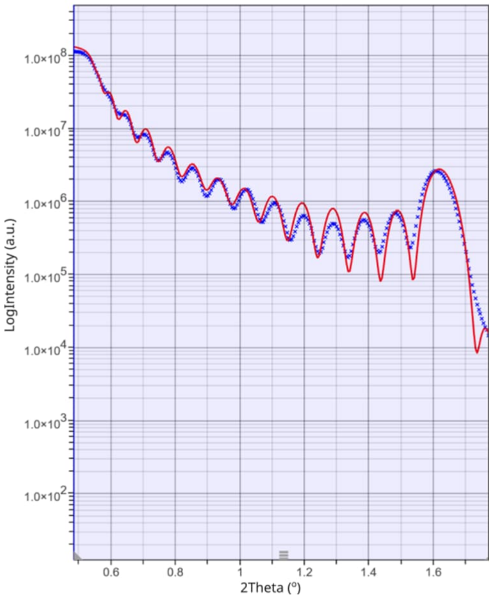

Figure S3. Representative XRR fitting for a TiO_2:PDA SL thin film (n = 14). Blue symbols correspond to acquired data, while red solid line corresponds to the fit. χ^2 achieved for this fit was 0.026592.

	HQ	TPA	A-TPA	PDA
Organic layer density (g cm^{-3})	1.74	1.44	1.41	1.59
Organic layer thickness (nm)	0.69	0.95	0.97	0.82

Table S2. Average organic layer characteristics deriving from the fitting of the XRR patterns for the TiO_2:organic SL thin films investigated in this study. Reported values resulting from the mathematical average related to each individual SL system with $3 \leq n \leq 56$.

Section III. *Optical properties of TiO_2 – organic SL thin films*

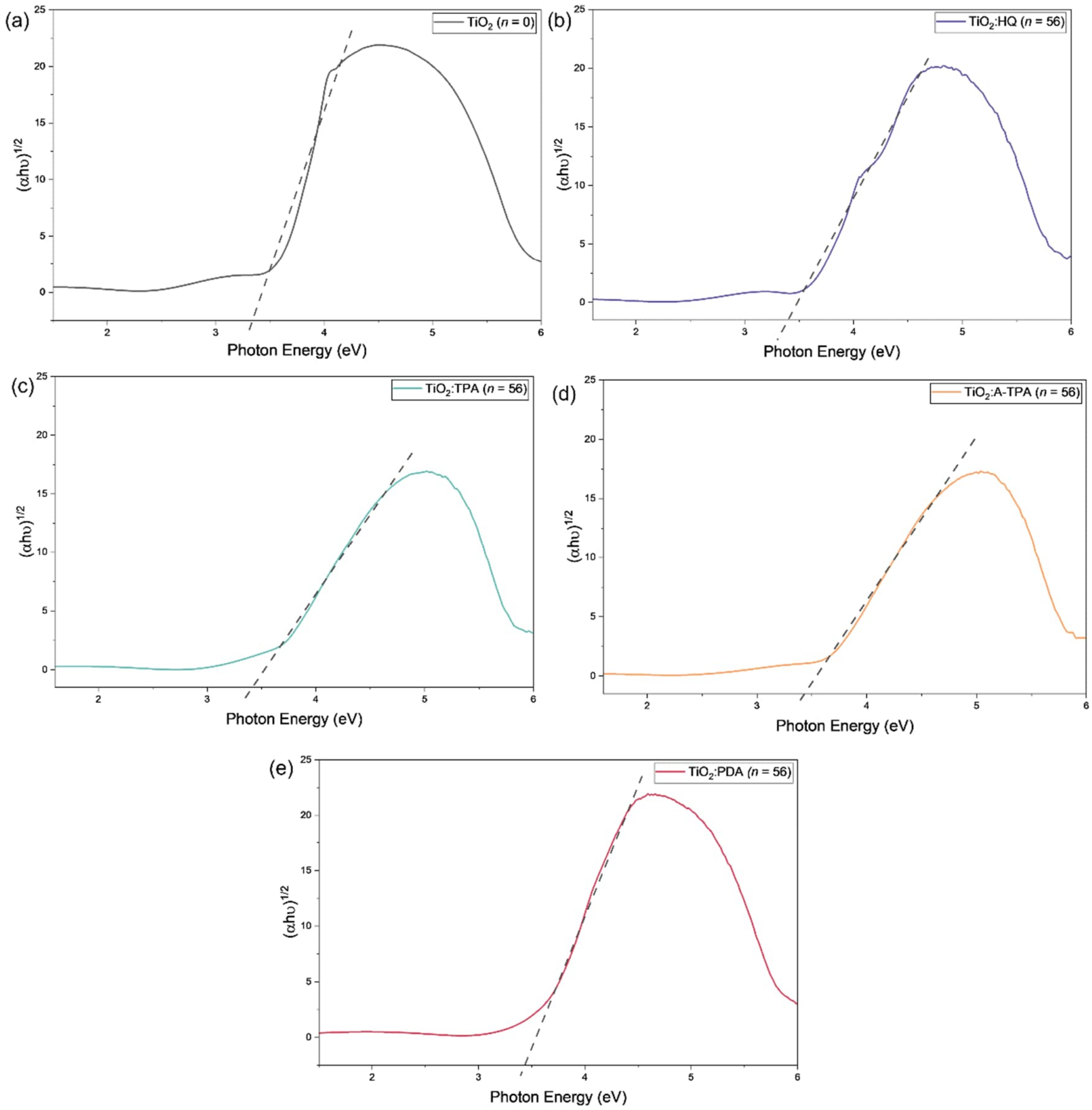

Figure S4. Tauc plot of $(\alpha h\nu)^{1/2}$ as a function of photon energy for (a) TiO_2, reference, and (b) TiO_2:HQ, (c) TiO_2:TPA, (d) TiO_2:A-TPA, and (e) TiO_2:PDA SL thin films with *n* = 56.

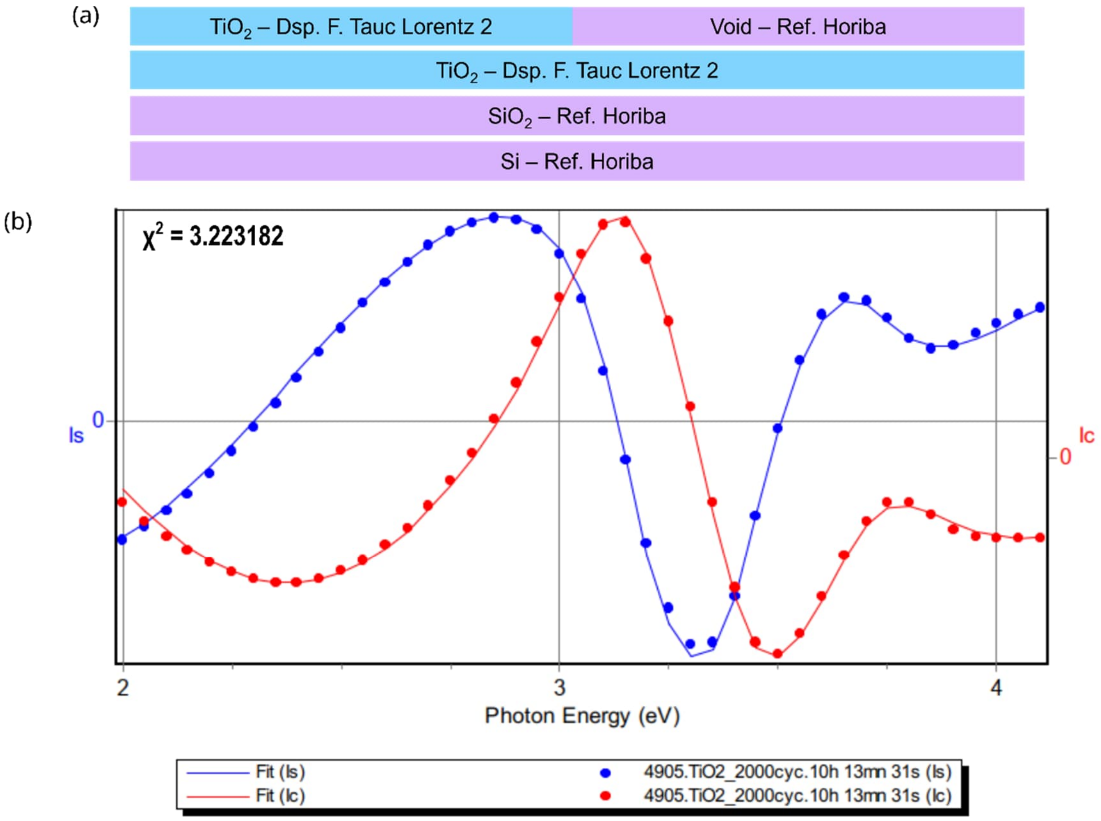

Figure S5. (a) Sample architecture employed in the SE model to fit the data of TiO_2 thin films, fit shown in (b). Initial thickness value is from the XRR data modelling. The "multiguess" function was employed to determine the individual layer thicknesses. This requires providing a nominal thickness value included in a wider thickness range as an initial guess to ensure maximum iterations of the fit inside this range (thus more freedom). It ultimately results in a more precise optimization of the thickness parameter compared to the case of a fixed nominal thickness value. Surface roughness is modelled according to the effective medium theory (EMT). Solid dots correspond to acquired data, while solid lines correspond to the fit. Measured data corresponds to the variables Is = sin(2Ψ) sin(Δ) and Ic = sin(2Ψ) cos(Δ), respectively shown in blue and red. χ^2 value is specified in the graph.

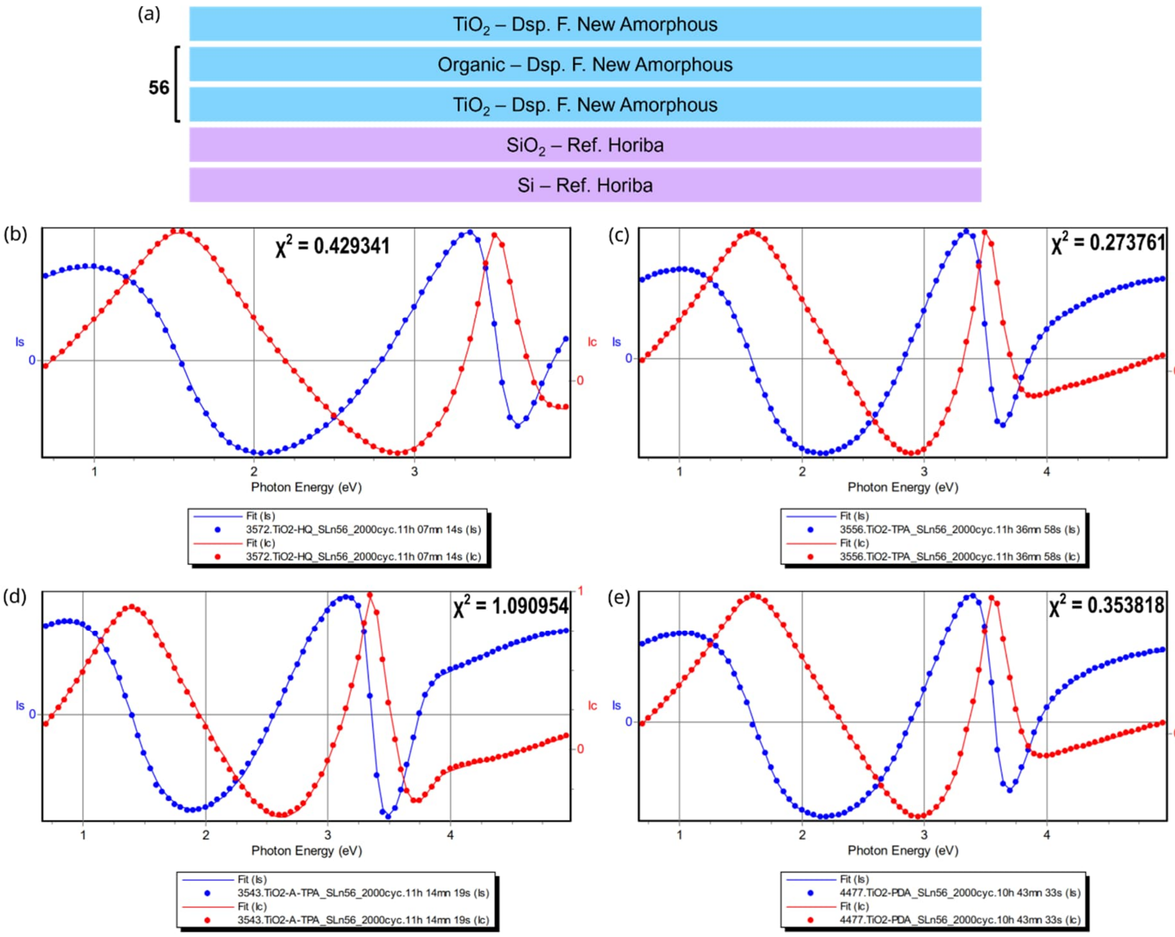

59,

Figure S6. (a) Sample architecture employed in the SE model to fit the data of TiO_2:organic SL thin films with n = 56. The value of n is set as the SL repetition of the respective TiO_2 and organic layers, with a final TiO_2 surface layer. Initial thickness values for each layer are from XRR data modelling. The "multiguess" function was employed to determine the individual layer thicknesses. This requires providing a nominal thickness value included in a wider thickness range as an initial guess to ensure maximum iterations of the fit inside this range (thus more freedom). It ultimately results in a more precise optimization of the thickness parameter compared to the case of a fixed nominal thickness value. SE data fitting for the n = 56 SL thin films of (b) TiO_2:HQ, (c) TiO_2:TPA, (d) TiO_2:A-TPA, and (e) TiO_2:PDA. Solid dots correspond to acquired data, while solid lines correspond to the fit. Measured data corresponds to the variables Is = sin(2Ψ) sin(Δ) and Ic = sin(2Ψ) cos(Δ), respectively shown in blue and red. χ^2 value for each case is specified in the respective graph.

Section IV. *Analysis of surface wettability of TiO2:organic SL thin films*

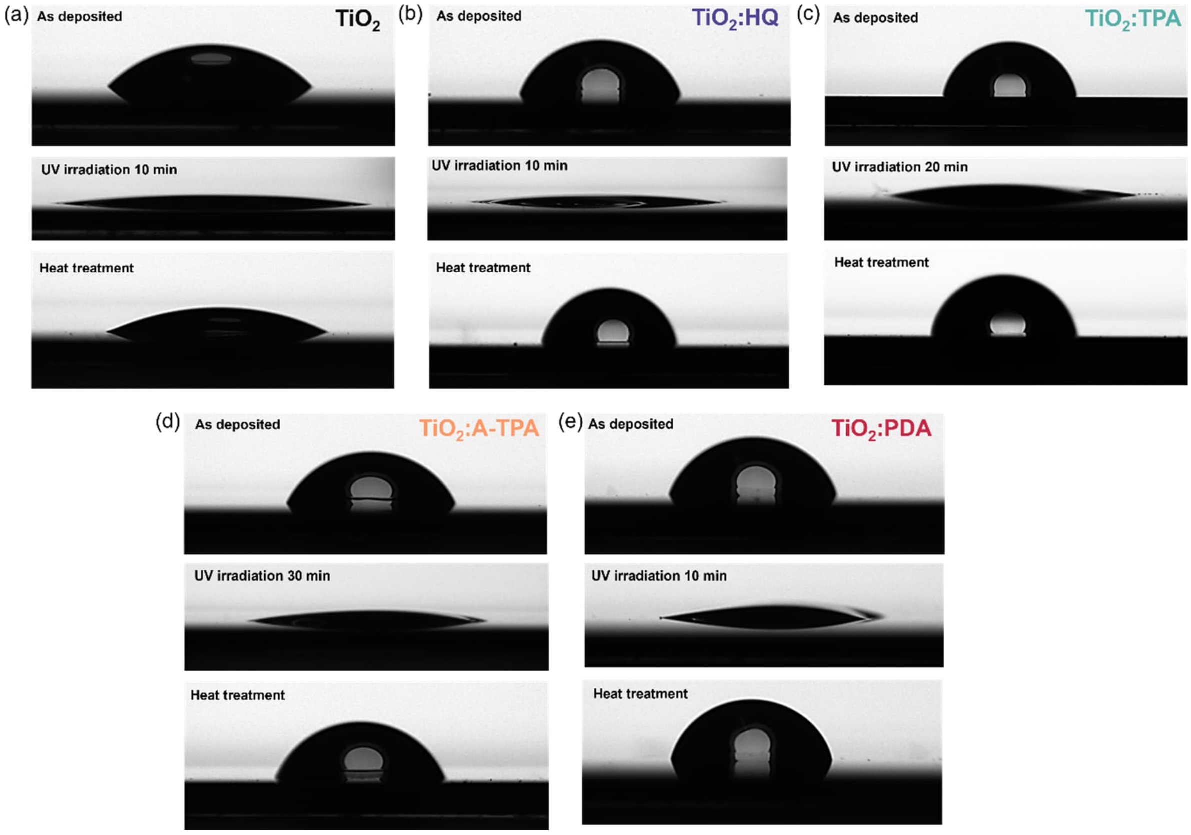

Figure S7. Recorded images of water contact angle measurement for (a) TiO_2 reference, and (b) TiO_2:HQ, (c) TiO_2:TPA, (d) TiO_2:A-TPA, (e) TiO_2:PDA SL thin films (n = 56) as deposited (upper panels), following UV irradiation (central panels), and after annealing at 100 °C (lower panels), showing the reversible character of the superhydrophilic surface behavior. The image recorded after UV irradiation corresponds to the final irradiation time for each thin film.

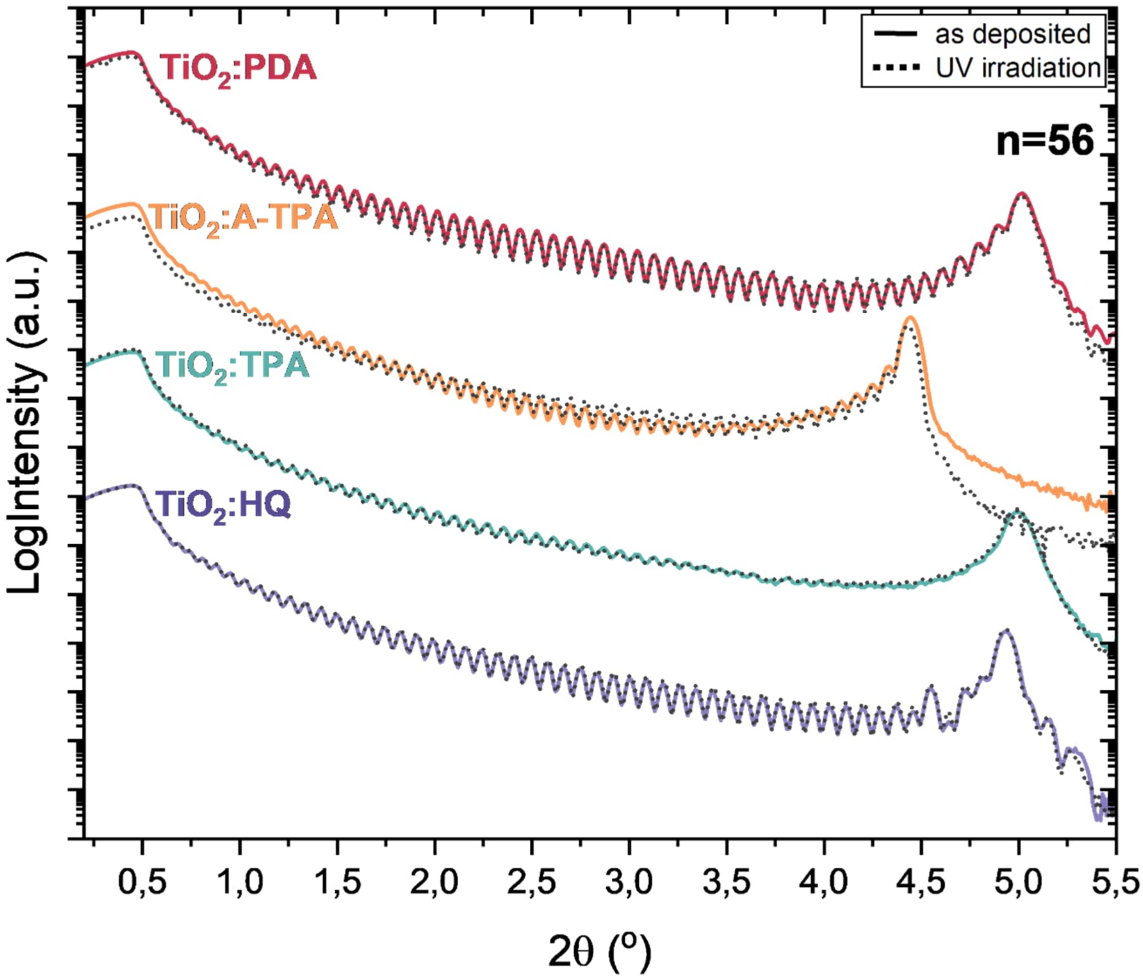

Figure S8. XRR patterns of the TiO_2:organic SL thin films shown in Figure 8 of the main text acquired as deposited (solid lines), and after UV irradiation (dotted lines, corresponding to the final irradiation time for each film) to confirm that no destructive effect occurs upon the UV irradiation on the SL architecture.

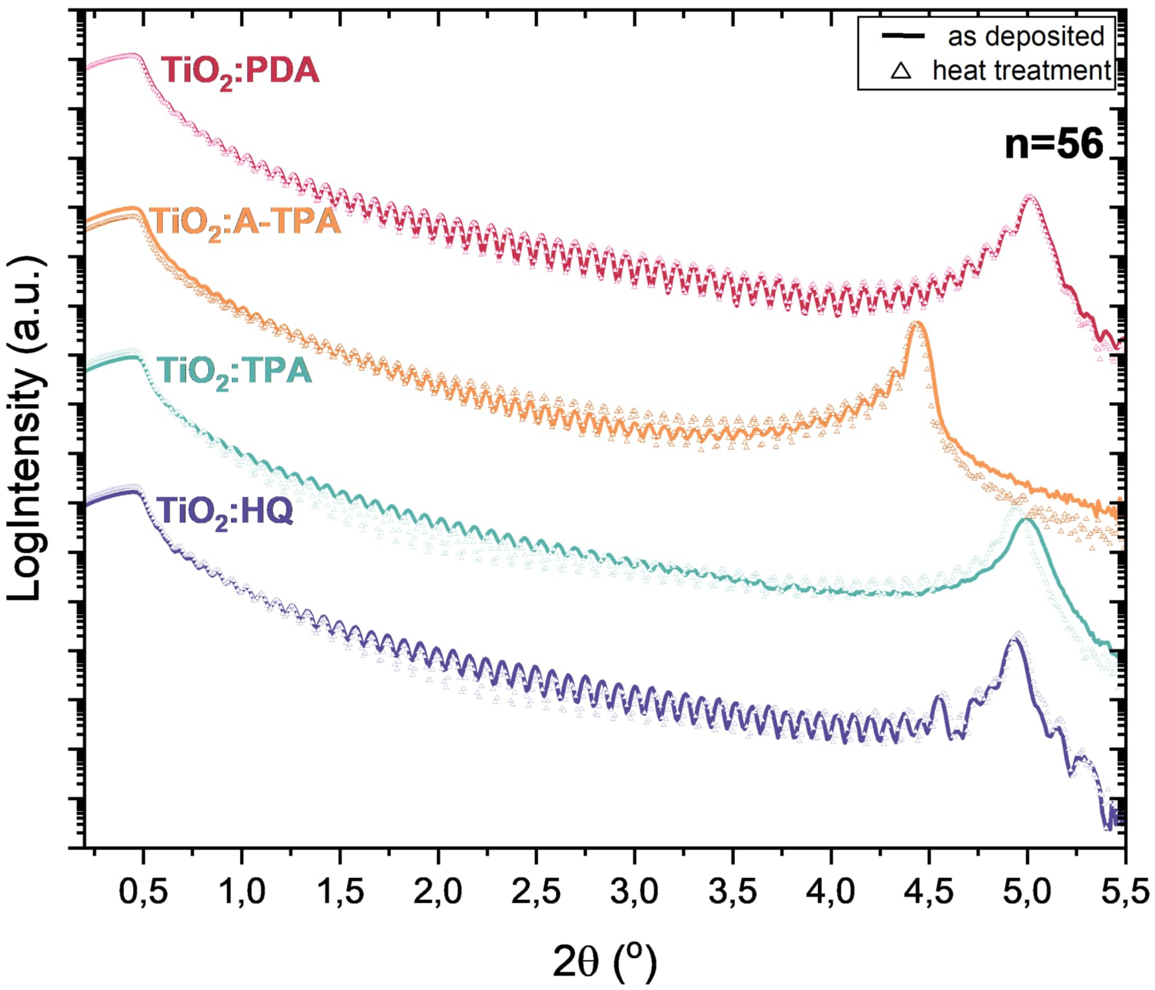

Figure S9. XRR patterns of the TiO_2:organic SL thin films shown in Figure 8 of the main text acquired as deposited (solid lines), and after UV irradiation and heat treatment (triangles, corresponding to the final irradiation time for each film), to confirm the thermal stability of the SL architectures.

Section V. *Summary of functional properties of TiO_2:organic SL thin films*

	TiO_2	TiO_2:HQ	TiO_2:TPA	TiO_2:A-TPA	TiO_2:PDA
**SL structural characteristics**					
Organic layer density [g cm^{-3}]	-	1.74	1.44	1.41	1.59
Organic layer thickness [nm]	-	0.69	0.95	0.97	0.82
**Optical properties**					
Optical band gap (SE) [eV]	3.20	3.21	3.35	3.42	3.32
Refractive index (λ = 633 nm)	2.513	1.885	1.585	1.989	1.989
**Mechanical properties**					
COS (%)	0.64 ± 0.12	-	0.91 ± 0.09	0.99 ± 0.17	0.77 ± 0.09
R_c (mm)	4.0 ± 0.7	-	2.8 ± 0.3	2.6 ± 0.5	3.25 ± 0.4
**Surface wettability**					
Water contact angle [°] (as deposited)	45.20 ± 1.25	73.53 ± 4.74	79.99 ± 1.63	65.72 ± 0.07	72.87 ± 1.16
Water contact angle [°] (UV irradiation)	9.93 ± 0.70	9.86 ± 1.49	10.92 ± 0.78	8.38 ± 0.11	6.23 ± 2.9
Water contact angle [°] (heat treatment)	25.37 ± 0.98	78.88 ± 0.78	83.70 ± 0.48	62.58 ± 0.41	76.54 ± 0.74

Table S3. Summary of the functional properties exhibited by the TiO_2:organic SL thin films.
